# Adaptive teaching assistance model combining generative AI and big data analytics

**DOI:** 10.3389/fpsyg.2026.1844703

**Published:** 2026-09-14

**Authors:** Zhiyun Zhu, Chen Luo, Lijuan Li

**Affiliations:** 1 Department of Global Communication Arts, Sunchon National University, Suncheon-si, South Jeolla, Republic of Korea; 2 College of Music, Myongji University, Seodaemun-gu, Gyeonggi-do, Republic of Korea; 3 School of Preschool and Primary Education, Shenyang Normal University, Shenyang, Liaoning, China

**Keywords:** big data analytics, generative artificial intelligence, multi-modal contrastive learning, music education assistant, reinforcement learning framework

## Abstract

The lack of personalized teaching resources and real-time evaluation in music education severely limits teaching effectiveness and cannot meet the diverse artistic expression needs of learners. Therefore, this paper proposes an adaptive teaching assistance model that combines generative artificial intelligence with big data analysis. This paper uses a cross modal transformer (CMT) to align performance audio with symbol score features, and employs a Proximal Policy Optimization (PPO) reinforcement learning framework to convert learners’ performance features into reward signals to generate dynamic content. At the same time, this paper conducted a quasi experimental technical validation for 12 weeks, involving 120 undergraduate students majoring in music (60 experimental students and 60 control students); the experimental group received feedback generated by the system, while the control group received traditional guidance. The results showed that the peak cosine similarity between the generated practice trajectory and the skill level of learners at the primary difficulty level was 0.962 ± 0.014, and the system’s error detection recall rate for complex rhythm errors was 91.2% ± 1.8%. After 300 training iterations, the cumulative expected return of the PPO mechanism remained stable at 95.12 ± 1.23 (average~0.79 per step). The learning trajectory of the experimental group was significantly steeper than that of the control group, as evidenced by the significant group time interaction [*β* = 0.52, 95% CI (0.31,0.73)], providing statistical support for the model’s potential to accelerate short-term skill improvement. These findings suggest that the proposed closed-loop system provides a promising algorithmic framework for personalized feedback in music education, but educational statements should be interpreted within the scope of testing conditions and technical proxy indicators reported.

## 1 Introduction

As digital transformation accelerates, music education is rapidly shifting from traditional face-to-face instruction to intelligent assistance. This transformation involves the use of generative artificial intelligence (AI) and big data analytics. However, it should not be seen as a simple “technological transplant” of how music has historically been taught. Instead, it requires a fundamental restructuring of how music is taught. Music education is not only about knowledge transmission. It also demands real-time interaction between teachers and students in aesthetic perception and practical skill development. Because music learning heavily relies on sensory experience, technology-assisted teaching has become essential to overcome instructional obstacles and provide highly personalized support to large groups of learners (Merchán Sánchez-Jara et al., 2024; Guetalla et al., 2024). This transformation is fundamentally supported by recent advancements in educational psychology and music learning theories. Effective music instruction relies heavily on deliberate practice and precise formative assessment, where the timing and specificity of feedback are crucial for motor-skill learning (Macnamara and Maitra, 2019). The integration of adaptive learning systems and human-AI collaboration further enhances motivation and engagement, fostering a foundation for learner agency and expressive development (Ouyang and Jiao, 2021). Recent developments in intelligent systems further highlight the potential of AI to reshape instructional paradigms (Singh et al., 2023); specifically, adaptive AI interactions originally utilized in behavioral and autism therapies demonstrate personalized feedback mechanisms that are highly transferable to music education.

Despite progress, several critical challenges remain. First, perception accuracy is still limited. For example, Kaori Kuromiya’s team found that previous methods could measure basic parameters such as loudness, but they failed to accurately describe the motion characteristics of timbre manipulation during touch. This leads to inaccurate feedback for artistic expression (Kuromiya et al., 2025). Second, environmental interference reduces recognition accuracy. Szwoch G confirmed that audio capture in multi-speaker systems (e.g., theatre sound systems) suffers from signal crosstalk, which lowers the accuracy of sound recognition (Szwoch, 2025). Third, emotional quantification is difficult. Shi L pointed out that traditional teaching methods are not effective in adapting to learners’ emotional states, whereas AI could improve emotional regulation for better language retention and engagement (Shi, 2025). Fourth, audio signal processing needs improvement. Chen M et al. noted that current digital audio processing methods lack resolution for complex audio signals, limiting accuracy and realism (Chen et al., 2024). Recent work on audio data augmentation and audio classification enhancement has shown promising results in improving model robustness under diverse acoustic conditions (Kumar et al., 2024; Kumar et al., 2020). Fifth, long-term structural coherence and subtle emotional variations over time remain problematic. Zhang M confirmed that existing models generate audio of significantly lower quality than human performance (Zhang, 2025).

In response, researchers have tried various technological paths, each with its own limitations. Recent advances in audio representation learning have also explored optimization strategies to bridge this quality gap (Park et al., 2020). Song G et al. reported that traditional hidden Markov models ignore beat frequency stability, resulting in redundant hidden states and high computational complexity, making it difficult to efficiently and accurately perceive music beat frequency (Song and Wang, 2022). Andrea et al. showed that convolutional neural networks (CNNs) can roughly locate notes on a staff, but their recognition accuracy is constrained by complex symbolic contexts and cannot meet high-precision pitch retrieval requirements (Andrea and Zahra, 2021). Tuo Q et al. achieved meaningful results in pop music style classification using support vector machines (SVM), but without enhanced music signal processing and effective separation of percussion and harmony components, high-precision recognition remains difficult (Tuo and Zhao, 2023). Barsha et al. used generative adversarial networks (GANs) to synthesize realistic artificial data, but they often suffer from mode collapse during training, which reduces data diversity (Barsha and Eberle, 2025). Hrishikesh Magadum’s team built a hybrid recommendation system that uses dimensionality reduction and similarity calculations to respond to evolving user preferences (Magadum et al., 2024). Although these methods have made progress in specific areas, they share a common problem: the disconnection between the “generation” and “analysis/evaluation” stages. Consequently, existing systems cannot form a real-time, dynamically evolving teaching loop. This gap highlights the urgent need to deeply integrate generation and evaluation capabilities.

Therefore, a new paradigm is required—one that integrates perception, analysis, generation, and feedback into a unified framework. This paper constructs a dynamic auxiliary model that combines multimodal contrastive learning and reinforcement learning. The model first collects students’ performance audio and multidimensional behavioral data to build a big data repository for teaching. Then, a multimodal contrastive learning transformer is used to model the correlation between audio features and symbolic music knowledge, achieving precise alignment of performance levels via cosine similarity loss. Based on this alignment, the analysis results are mapped into a reward space. The PPO algorithm is then applied to fine-tune the generation parameters in real time during interaction, so that the system’s output scores and accompaniments adaptively adjust to the learner’s progress. This design uses the inherent logic of technological integration—i.e., the output of the analysis stage drives the adjustment of the generation stage in real time, forming a reverse drive from evaluation to generation. It is designed to address the core challenges of perception accuracy and adaptive ability mentioned above. The system establishes an automated closed loop from monitoring to generation to intervention. The objective of this study is to provide a new algorithmic solution and technical architecture for improving resource adaptability and feedback flexibility in music education.

## 2 Method

### 2.1 Overall model architecture and fusion logic

The music education assistance model constructed in this study aims to achieve personalized teaching through a closed-loop path of “perception evaluation generation feedback”. The overall architecture of the model is shown in Figure 1, which achieves feature alignment of heterogeneous data in a unified high-dimensional semantic space through CMT collaborative processing of student audio and symbolic music input. The aligned feature vectors are synchronously transmitted to the big data analysis module and the generative AI module, where the big data analysis module quantifies performance into performance feature values through clustering and bias analysis. These quantified data are further mapped into real-time reward signals Rt and capability defect matrices Mt., which are used as external constraint logic inputs to the PPO framework. Driven by reward signals, the generative AI module utilizes a strategy network to optimize the parameter space and produce personalized exercise tracks that match the learner’s real-time learner skill progression. Ultimately, the generated teaching content is re applied to the input through a feedback loop, forming an adaptive teaching loop that operates in a closed-loop fashion.

**Figure 1:**
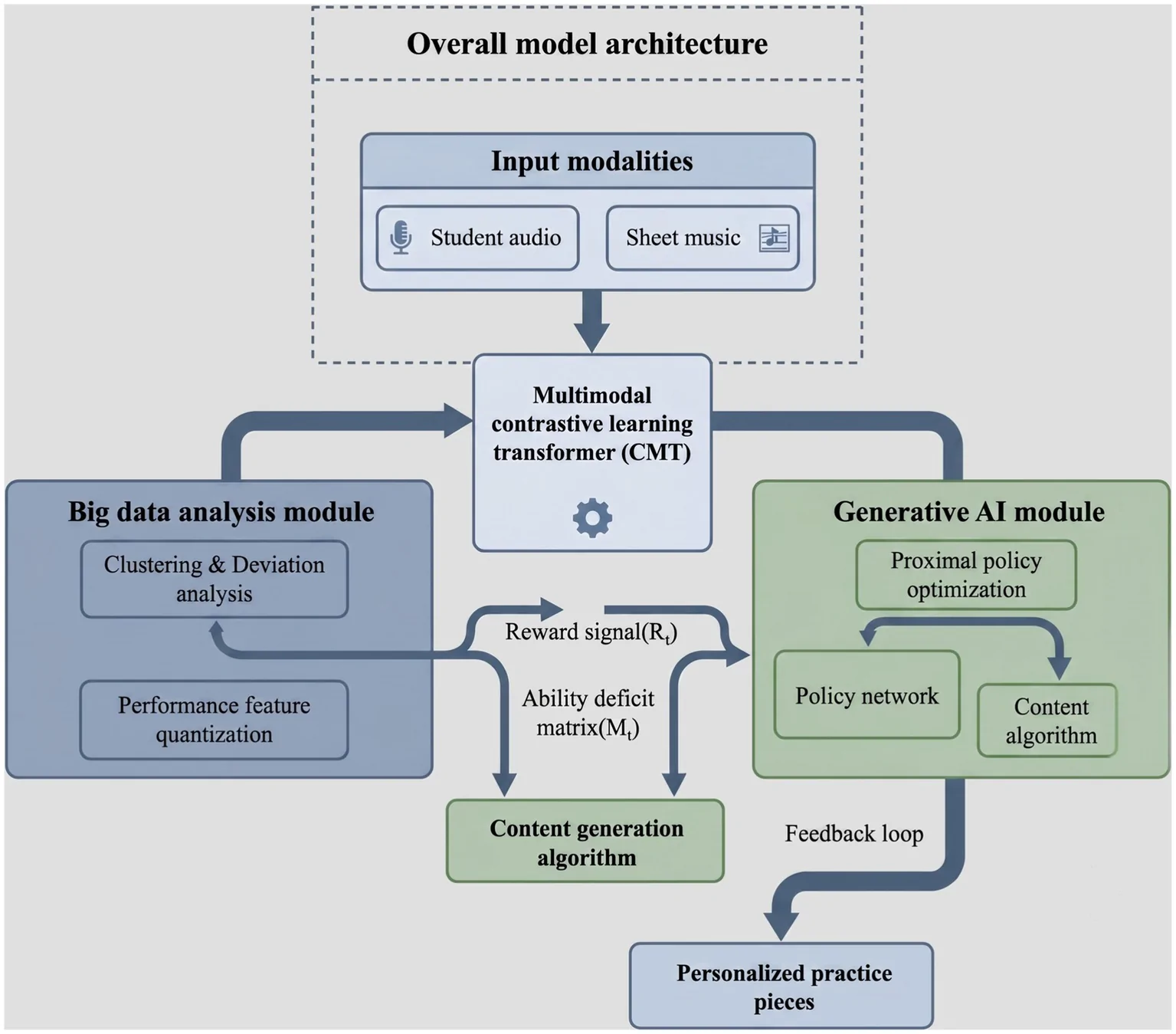
Overall architecture of music education auxiliary model combining generative AI and big data analysis.

### 2.2 Music feature extraction and alignment based on multimodal contrastive learning

In this study, the Cross-Modal Transformer (CMT) is chosen over traditional fusion methods (e.g., simple concatenation or additive attention) because it dynamically captures cross-modal correlations via the cross-attention mechanism. This allows the model to align audio and symbolic score features in a shared high-dimensional space while preserving temporal dependencies and artistic expression details. Compared to conventional approaches such as canonical correlation analysis (CCA), CMT offers greater flexibility in handling non-linear relationships and varying input lengths, which are common in music performance data.

In the stage of building the input layer of the model, the system synchronously receives the pulse code modulated audio signal recorded by the performance end and the standard digital music score text. For the audio modality, the model utilizes Mel Frequency Cepstral Coefficients (MFCC) technology to map the original time-domain waveform into a time-frequency representation with auditory perception characteristics, thereby filtering out environmental noise and preserving the core features of pitch and timbre (Zeng and Zeng, 2024; Turab et al., 2022). MFCC is selected because it closely mimics human auditory perception and has been widely validated for robust music feature extraction under noisy conditions (Marbun and Kodong, 2024). For the music score modality, the system uses word embedding techniques to transform notes, beats, and strong/weak symbols into high-dimensional dense vectors. The core processing logic relies on CMT, which achieves feature fusion between heterogeneous data through cross-attention mechanism (Ferraro et al., 2021; Nagrani et al., 2021). By drawing on highly robust multimodal architectures originally developed for complex tasks such as predictive healthcare analytics (Vavekanand et al., 2026), medical-image classification, and deepfake detection, this study adapts these high-precision, cross-domain representation alignment techniques to resolve the heterogeneous modalities of audio and symbolic scores in music education. This process involves a linear transformation of the query matrix Q, key matrix K, and value matrix V. By calculating the correlation weights between different modalities, precise alignment of the semantic meaning between the performance audio and the score is achieved. In order to quantify the consistency of two modalities in the latent space, the model introduces a contrastive learning loss function as a constraint, as shown in Equation (1):

$$L_{\text{contrast}}=-\sum_{i=1}^{N}\log\frac{\exp(\mathrm{sim}(a_{i},s_{i})/\tau )}{\sum_{j=1}^{N}\exp(\mathrm{sim}(a_{i},s_{j})/\tau )}\;$$ (1)

In Equation 1, $a_{i}$ represents the feature vector of the i-th audio clip, $s_{i}$ represents the corresponding score feature vector, sim represents the cosine similarity calculation function, τ is the temperature parameter that adjusts the smoothness of the distribution, and N is the size of the current batch. Contrastive learning has emerged as a powerful paradigm for learning aligned cross-modal representations without explicit supervision (Vavekanand and Kumar, 2025). By minimizing the loss function, the model drives audio and music scores with the same semantic content to approach each other in the feature space, while pushing unrelated samples away from each other. Effective integration of heterogeneous modalities is a central challenge in multimodal learning (Javed et al., 2025). In the feature fusion stage, the multi-head cross-attention mechanism dynamically captures the subtle fluctuations in intensity in the audio signal and the correlation between the strong and weak terms marked in the music score, and outputs aligned feature vectors with artistic expression dimensions. This alignment method provides a high-dimensional semantic reference for subsequent learning behavior diagnosis. The relevant parameter settings are shown in Table 1, and the feature extraction logic and attention mapping process are shown in Figure 2.

**Table 1:** Parameter configuration table for multimodal feature vector extraction.

Feature type	Processing method	Dimension/rate	Component	Alignment function
Audio modality	MFCC analysis	44,100 Hz	$a_{i}$ Vector	Cosine similarity
Score modality	Word embedding	512 Dim	$s_{i}$ Vector	Cosine similarity
Core layer	CMT architecture	1,024 Dim	Q, K, V Matrix	Cross-attention
Loss constraint	Contrastive learning	Batch Size N	Temp τ	Softmax scaling

**Figure 2:**
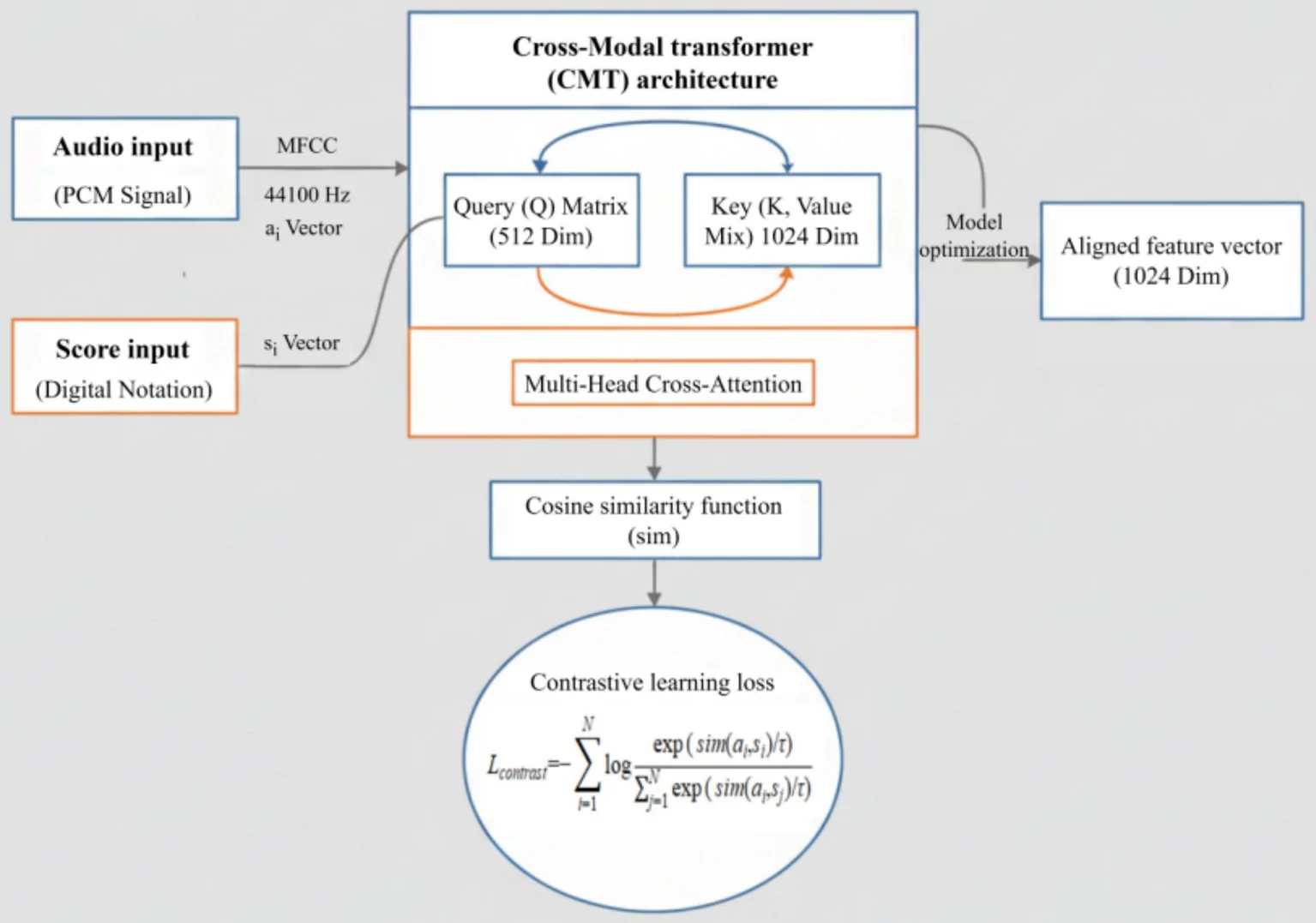
Multi-modal contrastive learning transformer feature alignment process.

Table 1 shows the quantization benchmarks for audio and score data during the input stage. The audio modality ensures the integrity of the original signal through a sampling rate of 44,100 Hz, and uses Mel frequency cepstral coefficients to analyze and produce a vector $a_{i}$ representing the measured features. The music score modality relies on 512 dimensional word embedding technology to map symbolic information to semantic vectors $s_{i}$ equivalent to audio features. The feature fusion of the core layer is achieved through a 1,024 dimensional multimodal contrastive learning transformer architecture, and a cross modal attention mechanism is constructed using linear transformations of query, key, and value matrices. The constraint logic of contrastive learning loss function is adjusted by batch size N and temperature parameter τ, combined with cosine similarity function and normalization scaling operator, to ensure the alignment accuracy and distribution stability of heterogeneous data in the hidden space.

Figure 2 depicts the evolution path of heterogeneous music data from raw input to semantic alignment. The data flow starts from the dual-mode parallel input terminal, and the PCM (Pulse Code Modulation) audio signal at a sampling frequency of 44,100 Hz is analyzed and converted into an audio feature vector $a_{i}$ through MFCC. In synchronization, the digitized music score information is mapped into a 512 dimensional music score feature vector $s_{i}$ through word embedding technology. These two sets of vectors with preliminary semantics flow towards the core CMT architecture. Under the action of cross-attention mechanism, the query matrix Q and the key value matrix K undergo nonlinear interaction and weight allocation. The multi-head attention structure dynamically captures the deep correlations between pitch, rhythm, and artistic expression dimensions. The fused information flow generates a 1,024 dimensional aligned feature vector, which is then fed into the cosine similarity calculation unit. Using the loss function $L_{\text{contrast}}$ , constraint and optimization of the samples is achieved through a comparison and learning process. The temperature (τ) and number of batch samples (N) jointly govern how the vectors in latent representation are distributed, whereby semantically similar audio and score features are drawn together, resulting in a high-accuracy model for aligning data with artistic semantics in a closed-loop architecture.

The detailed configuration of the CMT architecture is summarized in Table 2. Unless otherwise specified, these parameters were fixed across all experiments.

**Table 2:** Cross-modal transformer architecture configuration.

Parameter	Value
Number of layers	6
Number of attention heads	8
Hidden dimension	1,024
Feed-forward dimension	2048
Positional encoding	Sinusoidal
Activation function	ReLU
Dropout rate	0.1 (attention + FFN)
Normalization	Pre-norm LayerNorm
Total parameters	~48 M
Initialization	Xavier uniform
Sequence length (padded)	1,024
Padding strategy	Masked attention

MFCC served as the primary acoustic input to the CMT. CQT was computed only for auxiliary visualization (e.g., generating spectrogram images for instructor reference) and was not provided to the model; thus, all reported performance metrics reflect MFCC-based processing only.

### 2.3 Multidimensional evaluation of learning performance and mapping of reward signals

To quantify learner performance, we employ Dynamic Time Warping (DTW) for rhythm evaluation because it robustly handles local temporal misalignments between played audio and reference score, which are common in student performances. Gaussian Mixture Models (GMMs) are chosen for pitch accuracy assessment due to their ability to model multimodal error distributions (e.g., occasional octave jumps) and provide probabilistic uncertainty estimates. The weighted Euclidean distance is adopted as a unified metric because it allows domain-specific weighting of pitch, rhythm, and dynamics according to pedagogical importance.

From the data collected through this module and the clustering analysis, the technical execution level of the learners was characterised quantitatively (Christodoulou et al., 2024; Mehra et al., 2025).

The clustering of the audio data and the corresponding standard scores was performed using the DTW algorithm to measure the distance between the two datasets (Kraprayoon et al., 2024; Müller et al., 2021). This allowed for the identification of small deviations in the rhythmic dimension. For the evaluation of pitch, the system used the probability autocorrelation function to identify the fundamental frequency and subsequently compared this with the standard semitone frequency using GMMs (Gaussian Mixture Models). This provided a quantitative measure for the distribution characteristics of pitch error (Zheng, 2022; Cui, 2023).

To accurately measure the degree of deviation of individual performance characteristics from the ideal artistic indicators, the model would employ the weighted Euclidean distance metric (Bei et al., 2021; Gonzalez and Prati, 2023), which is represented by the Equation 2 shown in below.

$$D=\sqrt{\sum_{k=1}^{M}\omega_{k}{\left(x_{k}-{\hat{x}}_{k}\right)}^{2}}$$ (2)

Equation 2 defines the comprehensive evaluation score (specifically, the weighted Euclidean distance), where the parameters are as follows: D = Comprehensive evaluation score (weighted Euclidean distance), $\omega_{k}$ = the weight coefficient of the kth evaluation dimension, $x_{k}$ = the measured feature value of the learner in the defined evaluation dimension (i.e., pitch, rhythm or intensity), ${\hat{x}}_{k}$ = the standard benchmark of performance in this evaluation dimension, with M = total number of evaluation dimensions. The thresholds and weights in Table 3 were determined through a two-stage process. First, error thresholds (±15 ms for rhythm, ±10 cents for pitch, ±8 velocity levels for dynamics) were derived from expert consensus among three senior instructors with >10 years of teaching experience, who independently rated the perceptual acceptability of deviations in 50 pilot recordings spanning beginner to advanced levels. The reported thresholds represent the 90th percentile of errors judged as “acceptable” by at least two experts. These thresholds were held constant across instruments and proficiency levels, as the perceptual criteria for rhythmic stability and pitch accuracy are instrument-independent; however, for absolute beginners, a relaxed threshold (±20 ms, ±15 cents) is applied during the first 2 weeks of intervention to prevent over-correction.

**Table 3:** Quantitative benchmark for multidimensional evaluation indicators of learners.

Evaluation dimension	Calculation method	Error threshold	Weight coefficient ( $\omega_{k}$ )
Rhythm stability	DTW algorithm	±15 ms	0.35
Pitch accuracy	GMM fitting	±10 cents	0.4
Dynamic control	Velocity mapping	±8 levels	0.25
Skill shortfalls	Weighted Euclidean distance	—	—

Second, weights ( $\omega_{k}$ = 0.35, 0.40,0.25) were assigned using the Analytic Hierarchy Process (AHP) based on pairwise importance comparisons provided by the same three expert instructors. Pitch accuracy received the highest weight because fundamental frequency errors directly affect melodic recognition and are pedagogically prioritized in instrumental training; rhythm and dynamics follow in descending order. These weights were fixed across all experiments to maintain a consistent evaluation standard.

Before aggregation via the weighted Euclidean distance in Equation. 2, each dimension was normalized using min-max scaling to the range [0, 1] based on the training-set statistics, ensuring that all dimensions contribute on a common scale and preventing any single dimension from dominating due to its native unit magnitude.

The learner’s skill gap is determined through the offset of clustering center points and can produce a vectorized probability distribution matrix indicating the skill gaps (ability deficiencies) across the evaluation dimensions, which can be used directly in the subsequent generation process as a boundary condition for adaptive generation of teaching content, ensuring that the generated content covers the student’s deficient areas appropriately. Big data can be used for objective diagnosis of teaching models and quantitative benchmarking of the evaluation dimensions is shown in Table 3, whereas the learning behavior graph’s construction logic and feature space distribution is depicted in Figure 3.

**Figure 3:**
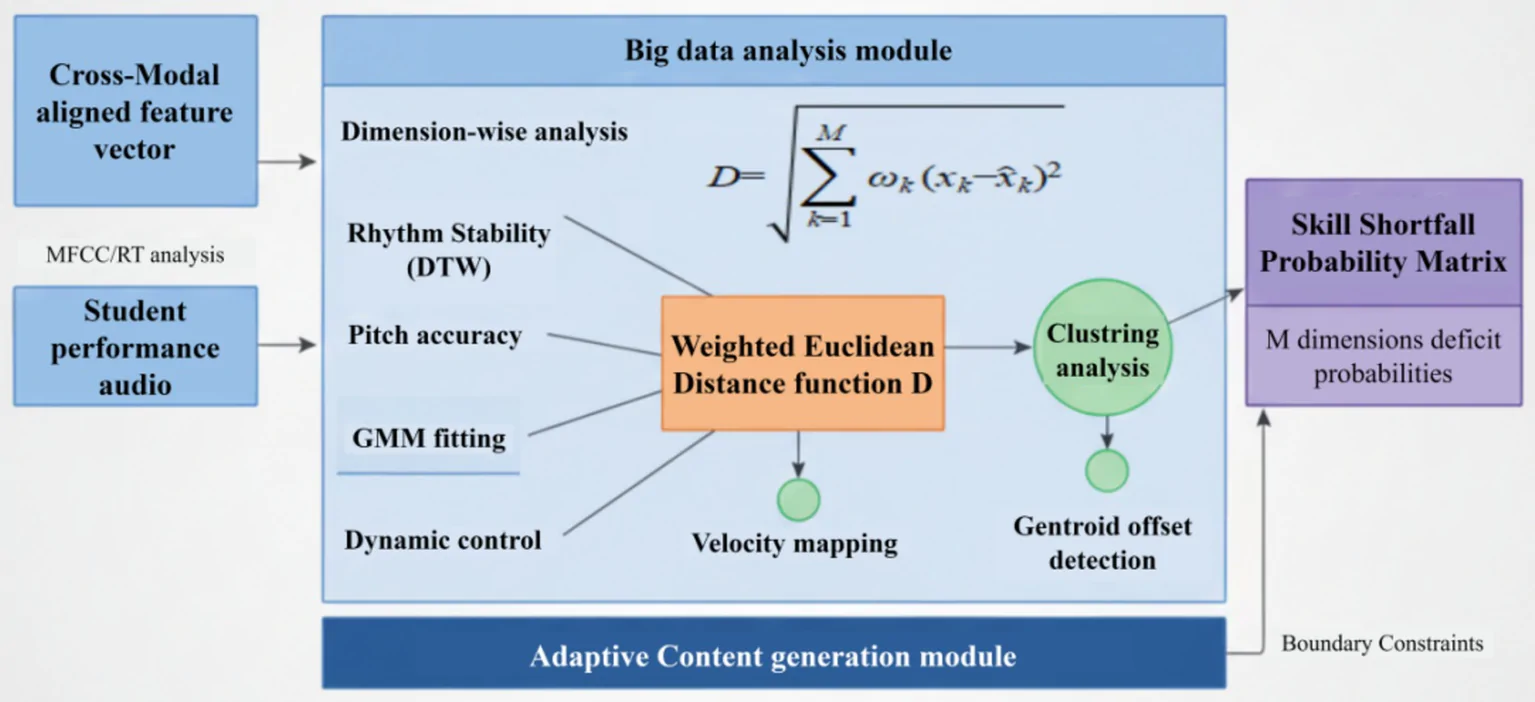
Spatial distribution of learning behavior graph based on clustering algorithm.

The criteria for feature mapping of performance data by the big data analysis module in Table 3 are as follows: The rhythm stability dimension uses the dynamic time warping algorithm to analyze the time value sequence. An error threshold of 15 milliseconds has been established for this dimension. The pitch accuracy dimension is based on the fundamental frequency characteristics of the pitch, and the measurement of pitch accuracy was performed using the Gaussian Mixture Model (GMM), with an error tolerance of 10 cents. The dynamic expression dimension captures amplitude variance through velocity mapping. All three dimensions are combined in a single evaluation system using weighted Euclidean distance to calculate the combined weight coefficients $\omega_{k}$ for each dimension, which are then used to create a two-dimensional probability distribution matrix that indicates the deviation between the measured feature value $x_{k}$ and the standard value ${\hat{x}}_{k}$ . With this benchmark, it is possible to objectively evaluate the deviation of the cluster center points from their original location to create a complete closed-loop logic framework that allows for further content generation Figure 4.

**Figure 4:**
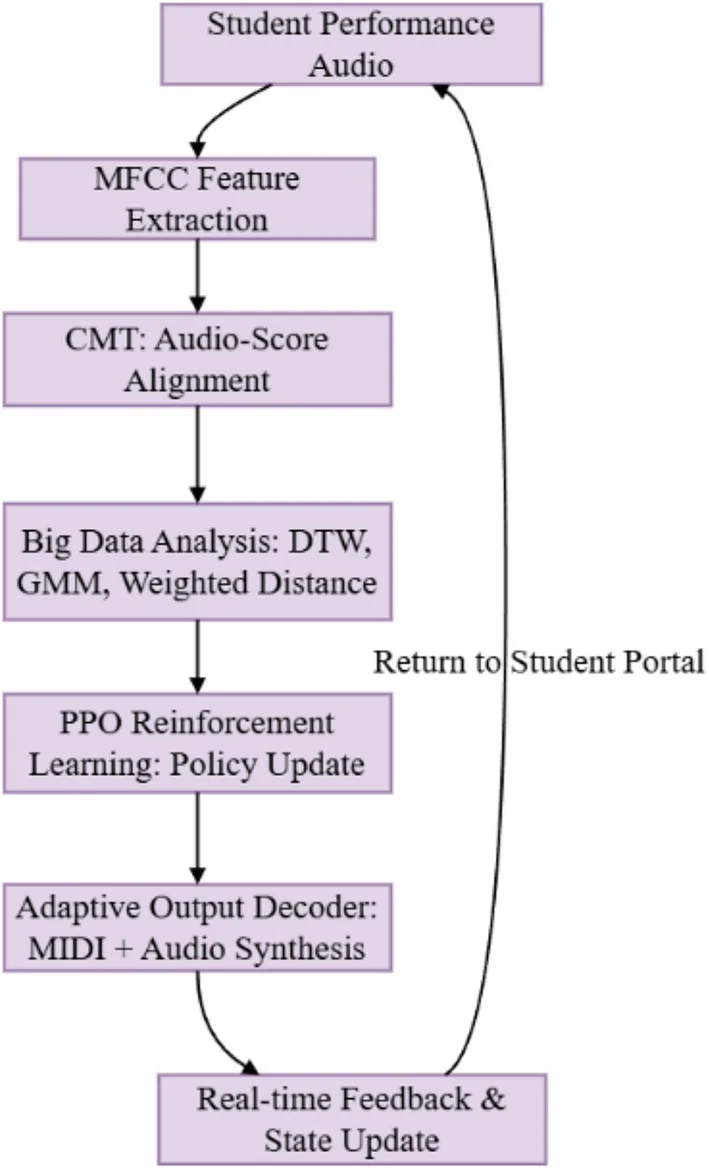
Simplified flow of the teaching-assistance closed loop.

The analysis process for measuring performance attributes (performance attributes in a multidimensional approach) and identifying areas where improvement is needed is shown in the figure. The measurement for a particular performance attribute, denoted $\omega_{k}$ , is processed using dynamic time warping algorithm, GMM fitting and velocity mapping to output three evaluation scalars; rhythm stability, pitch accuracy and strength expression. The outputs of these analyses (which are considered to be incompatible with one another) are then used as inputs into the core quantization unit, where they are aggregated together into a single comprehensive evaluation score by means of the weighted Euclidean distance and then combined with the weight coefficient $\omega_{k}$ . The resultant weighted feature vectors are mapped into the clustering space located within the center of the model, where the individual’s skills in any particular dimension are assessed by the distance from their current data point to the cluster center that corresponds to that performance attribute. Ultimately, the results of this diagnostic process are then converted into a probability distribution matrix of skill weaknesses produced at the right-hand side of the diagram and applied directly to the adaptive content generation module, which produces the required exercise tracks for each person along their personalized skill improvement paths.

### 2.4 Optimization of generative strategies based on PPO reinforcement learning

Proximal Policy Optimization (PPO) is selected over other reinforcement learning algorithms for several reasons. First, PPO provides stable and efficient updates by clipping the probability ratio, which prevents destructive policy changes—a critical requirement for maintaining musical coherence during iterative content generation. Second, PPO is simpler to implement and has lower computational overhead, making it suitable for real-time interactive teaching systems. Third, PPO naturally handles continuous action spaces, which aligns with the need to continuously adjust musical parameters (tempo, dynamics, and note density).

After receiving the skill deficiency probability matrix output by the big data analysis module, the generative network enters the iterative stage based on the PPO framework (Padhye et al., 2023; Islam et al., 2021). This process converts teaching evaluation indicators into reward functions for reinforcement learning, and guides the model parameters to shift towards improving teaching adaptability by calculating the probability ratio between the current generation strategy and the update strategy. In order to prevent the collapse of the artistic logic of the generated songs caused by excessive parameter updates, the system introduces a clipped-surrogate objective function and uses hyperparameter control strategy to update the step size, as shown in Equation. 3:

$$L^{\text{CLIP}}(\theta )=E_{t}[\min(r_{t}(\theta ){\hat{A}}_{t},\text{clip}(r_{t}(\theta ),1-\epsilon ,1+\epsilon ){\hat{A}}_{t})]$$ (3)

In Equation. 3, θ represents the parameter vector of the generative network, $r_{t}(\theta )$ represents the probability ratio of the new and old strategies, ${\hat{A}}_{t}$ is the estimated value of the advantage function, and ϵ is the clipping coefficient that limits the update range.

In order to further optimize the temporal logic of the generated content, the model introduces a value function to evaluate the potential learning gains of the current generation state, and achieves accurate estimation of reward expectations by minimizing the mean square error loss, as shown in Equation. 4:

$$\begin{array}{c} L^{\mathrm{VF}}(\theta )={\left(V_{\theta }(s_{t})-V_{t}^{\text{target}}\right)}^{2} \end{array}$$ (4)

In Equation. 4, $V_{\theta }(s_{t})$ is the predicted value of the value function in state $s_{t}$ , and $V_{t}^{\text{target}}$ is the actual observed cumulative reward target value.

Under the common constraint of the dual loss function, the generative network adjusts the weights of the hidden layers through backpropagation algorithm, so that the output practice instructions dynamically adjust the rhythm complexity and range span while maintaining the stability of music tonality. The update logic of the strategy gradient ensures a strong correlation between the model output and the learner’s skill gaps, as shown in Equation 5:

$$\nabla_{\theta }J(\theta )=E_{t}[\nabla_{\theta }\log\pi_{\theta }(a_{t}|s_{t}){\hat{A}}_{t}]\;$$ (5)

In Equation. 5, J(θ) is the objective function of policy performance, and $\pi_{\theta }(a_{t}|s_{t})$ represents the probability distribution of taking the generative action $a_{t}$ in state $s_{t}$ . This mechanism realizes the underlying driving force of data analysis results on the generation algorithm, enabling the system to have a closed-loop optimization ability to automatically evolve teaching difficulty based on feedback signals, thereby ensuring the gradual progression of exercise content at the algorithm level.

The reinforcement learning environment was defined as follows. State $s_{t}$ consisted of the learner‘s current performance feature vector $x_{k}$ ​ (pitch, rhythm, and dynamics) concatenated with the difficulty level of the current exercise. Action $a_{t}$ was a three-dimensional continuous vector controlling tempo multiplier (0.7–1.3), dynamic range compression (0.5–1.5), and note density factor (0.6–1.4). The per-step reward function was defined as:

$$\begin{array}{c} R_{t}=-D_{t}+\alpha \cdot \Delta D_{t} \end{array}$$ (6)

In the Equation. 6, $D_{t}$ is the weighted Euclidean distance at time step t (each step corresponds to 30 s of practice), $\Delta D_{t}\;$ = $D_{t-1}$ Dt (i.e., improvement relative to the immediately preceding step), and α =0.3 balances current accuracy against progress. Thus, $R_{t}$ is computed at every time step (every 30 s), with each $R_{t}$ ranging from −10 (substantial deterioration) to +10 (substantial improvement). An episode corresponds to one 60-min session, consisting of 120 time steps. The episodic return is the sum of per-step rewards over the 120 steps. The discount factor was γ = 0.99. GAE used λ = 0.95. PPO clipping coefficient ϵ = 0.2 (defining the interval [0.8, 1.2] for the probability ratio). Entropy coefficient was 0.01, and value-loss coefficient was 0.5. Each PPO update used 10 epochs with mini-batch size 64. The policy and value networks shared a two-layer MLP (256–128 units, ReLU) with separate output heads. Training stopped when validation reward did not improve for 30 consecutive episodes. Exploration used a Gaussian policy with adaptive standard deviation (annealed from 0.3 to 0.05).

This cumulative reward value (95.12) represents the sum of episodic rewards accumulated over a single evaluation run (mean across 3 seeds, as reported in Section 4.3). Because each episode yields a reward in the range [−10, +10], the theoretical maximum for 120 steps per episode is +1,200 per session. The observed value of 95.12 thus indicates that the policy achieved a per-step average reward of approximately 0.79, which corresponds to consistently positive progress (reduction in D) across the session. Pedagogically, this means the generated exercises were consistently matched to the learner’s skill level, leading to measurable improvement in pitch, rhythm, and dynamics without task disengagement. For comparison, a random policy baseline (uniform action sampling) yielded a cumulative reward of 12.3 ± 4.7 under the same evaluation conditions, confirming that the PPO-driven policy substantially outperforms chance. The random baseline was computed over the same 120-step episode length, with actions sampled uniformly from the valid action space at each time step, aggregated across 30 independent evaluation runs (10 per random seed).

### 2.5 Adaptive output logic of dynamic teaching path

In the adaptive output stage of the dynamic teaching path, the system utilizes a deep learning decoder to transform the optimized strategy parameters into a multidimensional teaching resource stream. This process relies on a recursive neural network decoding architecture with temporal memory capability, which achieves synchronous mapping of Musical Instrument Digital Interface (MIDI) sequence and waveform synthesis parameters by receiving implicit vectors generated by the strategy (Rosalina, 2024; Mon, 2025). The state update of the decoder at each moment follows a conditional probability distribution, and by introducing an attention based mask decoding algorithm, it ensures that the generated melody lines maintain a high degree of consistency in tonal logic and rhythmic form. In order to achieve millisecond level real-time interactive response, the system constructed a memory buffer based on zero copy technology, decoupling and parallel processing the audio rendering engine and the interactive music score rendering engine. The output end adopts a parameter smoothing algorithm based on Kalman Filter to predict and correct the generated control signal, in order to eliminate the delay caused by network fluctuations. The adaptive degree of real-time output is controlled by calculating the dynamic feedback gain (Deepika and Rajitha, 2022; Seidel et al., 2024), as shown in Equation. 7:

$$\begin{array}{c} G(t)=K_{p}e(t)+K_{i}\int_{0}^{t}e(s)\mathrm{ds}+K_{d}\frac{\mathrm{de}(t)}{\mathrm{dt}} \end{array}$$ (7)

In Equation. 7, G(t) represents the adaptive adjustment gain at the current time, e(t) represents the deviation function between the learner’s real-time performance and the target benchmark, $K_{p}$ , $K_{i}$ and $K_{d}$ are proportional, integral, and derivative control coefficients, respectively, and s is an integral time variable used to characterize the influence of the learner’s historical performance on the current gain. This control logic feeds back the dynamic errors in student performance to the decoding layer, and adjusts in real-time the tempo, dynamics, and density of the generated accompaniment, as well as the density of the score prompts.

In order to ensure the coherence of the output content, the system introduces a state transition probability matrix to smooth the teaching advancement path. By maximizing the transition probability between the current teaching state and the historical learning path, the optimal output mode for the next stage is determined, as shown in Equation. 8:

$$\begin{array}{c} P(S_{t+1}|S_{t}|A_{t})=\frac{\exp(Q(S_{t},A_{t}))}{\sum_{a'A}\exp(Q(S_{t},a^{\prime }))} \end{array}$$ (8)

Equation. 8 contains four variables, where $S_{t}$ represents the current teaching state vector and $A_{t}$ indicates what the system has chosen as its action (as defined by a choice made by the learner). $Q(S_{t},A_{t})$ Provides an indicator of quality between S and A; for example, $Q(S_{t},A_{t})$ can determine if S is responding positively or negatively. A represents all available teaching interventions that can be used. The adaptive output mechanism such as the one discussed in this example takes the static characterization of a teaching exercise and allows the process to take on dynamic characteristics based upon how well the learner is responding to the exercises. Feedback loops provide information from the interaction back to the reinforcement learning layer on a millisecond by millisecond basis, which enables the complete re-shaping of the learning path through continuous reinforcement strategy tuning that can ultimately lead to the development of a learner experience that aligns with the fine-tuning of the reinforcement learning. A closed loop of automated processes from parameter injection through result feedback through the reconstruction of the parameters provides complete alignment of the learner’s learning experience with the learner skill progression.

To facilitate reproducibility and clarity of the proposed adaptive teaching loop, a simplified flowchart is provided below. This diagram highlights the main data flow and decision points.

The loop operates as follows: ( 1 ) student performance audio is captured and converted to MFCC features; ( 2 ) CMT aligns audio features with the symbolic score; (3) the big data analysis module quantifies errors in rhythm, pitch, and dynamics, producing a reward signal; (4) PPO updates the generation policy to maximize cumulative reward; (5) the decoder synthesizes a new exercise track; (6) the student plays the new track, closing the loop.

## 3 Experimental design and environmental configuration

### 3.1 Experimental dataset and preprocessing instructions

This research integrated heterogeneous data from various sources during the experimental preparation phase, to provide dataset resources to enhance both pre-training and fine-tuning of the model. For initial pretraining of the CMT’s feature extraction layers, we used the MAESTRO (MIDI and Audio Edited for Synchronous Tracks and Organization) dataset v3.0.0, available at https://magenta.tensorflow.org/datasets/maestro (licensed under CC BY-NC-SA 4.0). This corpus contains approximately 200 h of solo piano performances (1,276 MIDI-aligned audio recordings) spanning classical, jazz, and popular styles, recorded in studio and concert hall environments. It contains no copyrighted commercial recordings and is explicitly released for academic research. Importantly, pretraining on solo piano data is sufficient to learn fundamental timbral and structural representations (e.g., pitch contour, rhythmic stability, dynamic gradation) that transfer effectively to other tonal instruments such as violin, flute, and guitar, because the CMT learns cross-modal alignment at the score-feature level rather than instrument-specific timbre. There is no participant, session, or musical excerpt overlap between this pretraining corpus and our newly collected student dataset described in the remainder of this section. The MAESTRO data were used solely to initialize the foundational layers; no samples from this corpus were used in the validation, test, or educational-outcome analyses of our study. Due to the unique circumstances of an educational environment, this study developed an additional repository for data output from the implementation of customized teaching methods developed specifically for this study. The audio stream from students as they performed practice routines and the corresponding standard score comparison data were recorded in real-time, thus creating a customized instructional dataset.

To prevent temporal leakage and circularity, all model components were finalized before the 12-week educational intervention began. Specifically, the CMT feature extraction layers were pre-trained on the MAESTRO v3.0.0 dataset and then frozen, while the PPO generation policy was pre-trained offline and fixed for the entire intervention period. Additionally, the participant-level split into training, validation, and test sets was performed immediately after baseline assessment (Week 0) and sealed before any intervention data were collected. During the 12-week intervention, the system operated solely in a fixed-policy inference mode—no participant data were used for model updates or fine-tuning. All recordings were stored for post-intervention offline evaluation only.

The dataset comprised recordings from 120 undergraduate music majors (60 experimental, 60 control) over a 12-week period. During each weekly session, each participant recorded multiple short practice excerpts focusing on assigned technical exercises and repertoire passages; each excerpt lasted approximately 30 s on average. This resulted in approximately 4,320 audio recordings (120 participants × 12 weeks × 3 excerpts per session = 4,320) with a total duration of 2,160 min (4,320 × 0.5 min). All 120 participants completed the 12-week study, with no dropouts or exclusions. Importantly, throughout this 12-week period, the system’s core parameters remained frozen; no online learning or policy updates were performed based on participant interactions. Instruments included piano (*n* = 48), violin (*n* = 32), flute (*n* = 24), and guitar (*n*= 16). All practice excerpts for piano and guitar participants were restricted to monophonic melodic lines to maintain compatibility with the current audio processing pipeline. Epertoire covered Baroque (30%), Classical (35%), Romantic (20%), and Contemporary (15%) styles. Each recording was annotated by two expert instructors for pitch accuracy, rhythmic stability, and dynamic control, with inter-annotator agreement *κ* = 0.87. To clearly differentiate between technical model validation and educational outcome evaluation, we established a two-tier data usage protocol. For the educational outcome analysis, the entire cohort of 120 participants was included to provide sufficient statistical power for detecting learning gains. However, for the rigorous, unbiased evaluation of the model’s core algorithmic performance, we reserved the independent test set. This test set consisted of 18 participants (9 experimental + 9 control), representing a 15% hold-out split from the full dataset, who were entirely unseen during the model training (70%, *N* = 84) and validation (15%, *N* = 18) phases. Recordings from the same participant appeared in one partition only (no within-participant cross-partition leakage). The test-set participants were used for all final technical metric reporting. Feature normalization (min-max scaling) was performed using only the training-set statistics; these statistics were then applied to normalize validation and test data, ensuring no information leakage from the test set into preprocessing.

Further details regarding the experimental design are provided here. Participants were 120 undergraduate music majors recruited from Sunchon National University via course announcements between March and May 2025. Inclusion criteria: (1) current full-time music major, (2) principal instrument among piano, violin, flute, or guitar, and (3) ≥ 3 years of formal training on that instrument. Exclusion criteria: prior exposure to AI-based tutoring systems. Age ranged 20–24 (21.8 ± 1.4); mean prior training was 5.2 ± 1.8 years. Baseline proficiency (week 0 standardized test, grade 6 excerpt) showed comparable D-scores between groups: experimental 52.37 ± 6.81, control 53.04 ± 7.12 (*p* = 0.58). Instrument distribution was balanced: 24/16/12/8 (piano/violin/flute/guitar) per group.

Participants were allocated via stratified assignment (by baseline proficiency and instrument) by the primary investigator. Both groups were taught by the same two instructors, who received 2-h system training before the intervention. Both groups attended one 60-min session per week for 12 weeks (total 12 h). The control group received traditional teacher-led verbal feedback; the experimental group received the same repertoire and practice requirements but used the adaptive system for real-time feedback and generated exercise tracks. All other conditions were identical. Attendance was 100% (no withdrawals). Performance assessors were blinded to group assignment (recordings anonymized and randomized). Intervention fidelity was monitored via system logs and classroom observation; cross-group contamination was minimal due to headphone-based individual practice.

To prepare the audio for the algorithm, the first step in the preprocessing process is to denoise the audio using spectral subtraction. The next step is to convert the audio time series into a CQT (Constant-Q Transform), which creates an image-like feature map of the audio (geometric frequency distribution) that represents the logarithmic frequency characteristics of the audio signal. Digital audio score representations are then transformed to Event Encoding Sequence Format (ESEF) to establish logical correspondence between time-based events and audio frame data. To adjust for timing differences between the audio being played and the standard score timing, Dynamic Time Warping (DTW) alignment is performed on the audio and the score events to create a matched trajectory. The audio and symbolic-score preprocessing pipeline was parameterized as detailed in Table 4.

**Table 4:** Audio and symbolic-score preprocessing parameters.

Category	Parameter	Value
Audio format	File format/bit depth	16-bit PCM WAV
	Sampling rate	44.1 kHz
	Denoising	Spectral subtraction (over-subtraction 2.0, noise floor 0.001)
	Silence removal	Leading/trailing > − 40 dBFS for 0.2 s
	Loudness normalization	Not applied
MFCC	Number of coefficients	13 + delta + delta–delta = 39
	Window size/hop length	25 ms/10 ms
Symbolic score	Original format	MIDI/MusicXML
	Tokenization	Event Encoding Sequence Format (ESEF)
	Vocabulary size	512
	Event tuple	[pitch, onset, duration, velocity, articulation]
	Special tokens	Rests, meter

For time-series alignment and pitch modeling, DTW used a Sakoe-Chiba band with a window width of 200 ms, and GMM fitting employed three Gaussian components with diagonal covariance matrices, initialized via K-means.

The basis of this alignment is to minimize the total distance between the audio frame and the corresponding score events, as shown in Equation. 9.

$$\begin{array}{c} D(i,j)=d(q_{i},c_{j})+\min\{D(i-1,j),D(i,j-1),D(i-1,j-1)\} \end{array}$$ (9)

In Equation. 9, D(i,j) represents the cumulative distance value at position (i,j) in the alignment matrix, and $d(q_{i},c_{j})$ is the Euclidean distance between the i-th point of sequence q and the j-th point of sequence c.

To further enrich the training data diversity, additional augmentation strategies—including information-preserving approaches—have been explored in recent work (Kumar et al., 2023; Kumar et al., 2024). After completing the alignment, the data is normalized to the maximum and minimum values, and the feature values are linearly mapped to a closed interval of 0 to 1, thereby eliminating the magnitude differences between different acquisition devices, as shown in Equation (10):

$$\begin{array}{c} x^{\prime }=\frac{x-x_{\min}}{x_{\max}-x_{\min}} \end{array}$$ (10)

In Equation (10), $x^{\prime }$ is the normalized eigenvalue, x is the original input data, and $x_{\max}$ and $x_{\min}$ are the maximum and minimum values within the feature dimension, respectively. For reproducibility: data were split into training/validation/test (70/15/15) at participant level; random seed 42 was fixed; 5-fold cross-validation repeated 3 times. The processed experimental data meets the training requirements in terms of distribution balance and annotation accuracy.

To formally test whether the adaptive teaching model led to differential skill improvement over time, we employed a linear mixed model (LMM) for the educational outcome analysis (Section 4.6). The dependent variable was the comprehensive evaluation score D measured weekly for 12 weeks. The fixed effects included Group (experimental vs. control), Time (week, treated as a continuous variable from 0 to 12), and the Group × Time interaction. Baseline proficiency, measured by the D score at week 0, was included as a covariate to control for initial differences. To account for the non-independence of repeated measurements from the same participant, we included a random intercept for each participant. The primary statistical evidence for an accelerated learning trajectory was the coefficient of the Group × Time interaction (*β*) and its 95% confidence interval (CI). A positive β with a 95% CI excluding zero would indicate a significantly steeper improvement rate in the experimental group compared to the control group. All models were fitted using restricted maximum likelihood (REML) estimation. Effect sizes were calculated as Cohen’s *f*^2^ for the interaction term.

All participants provided written informed consent prior to enrollment. The study employed a quasi-experimental design: the experimental group (*n* = 60) received the proposed adaptive teaching assistance model, while the control group (*n* = 60) received traditional instruction. Both conditions were integrated into routine teaching activities, and all participants completed their regular practice schedule without additional class time. The study was conducted in accordance with the Declaration of Helsinki.

### 3.2 Experimental benchmark model and software and hardware environment

To validate the accuracy of the proposed architecture on challenging music education tasks through experimentation, the evaluation of the architecture was done through a series of tests conducted on high-performance computing clusters. All test information was gathered and analyzed through the same high-performance computing cluster so that no difference in hardware performance can affect either the runtime or the convergence rate of the tests. Additionally, the usage of structural similarity index and cross-entropy loss enabled the testing of both the training and testing stages of the architecture to evaluate the performance of the architecture during the training process. The logarithm loss approach shown in Equation (11) quantifies how closely the predicted outputs match the actual or ground-truth outputs of training data and thus allows for optimizing the weights of the model through the training stage of the architecture.

$$\begin{array}{c} L_{\mathrm{CE}}=-\frac{1}{N}\sum_{i=1}^{N}[y_{i}\log({\hat{y}}_{i})+(1-y_{i})\log(1-{\hat{y}}_{i})] \end{array}$$ (11)

In Equation (11), $L_{\mathrm{CE}}$ represents cross entropy loss, N is the size of the current training batch, $y_{i}$ represents the annotated labels in real teaching scenarios, and ${\hat{y}}_{i}$ is the predicted generation action or evaluation probability of the model. In the parameter optimization stage, the system uniformly adopts the Adam optimizer, which accelerates the convergence process of the loss function by adaptively adjusting the learning rate. The weight initialization of each benchmark model follows a normal distribution during the initial training stage. At the hardware level, high-performance computing cores with large-scale parallel processing capabilities are configured, coupled with highly integrated deep learning frameworks and audio processing modules, to ensure the throughput efficiency of massive music big data in feature extraction and strategy iteration processes. The specific hardware configuration parameters and software operating environment parameters are shown in Table 5.

**Table 5:** Hardware configuration and software environment parameters.

System component	Technical implementation	Core function	Calculation operator	Performance indicator
Compute hardware	High-performance cluster	Parallel processing	Batch size	Throughput efficiency
Software framework	Deep learning engine	Feature extraction	Adam optimizer	$L_{\mathrm{CE}}$ convergence
Audio module	Signal processing	Audio evaluation	Log-loss mapping	Predictive probability ${\hat{y}}_{i}$

The detailed specifications are as follows: Compute hardware: 4 × NVIDIA A100 (80 GB) GPUs, 2 × AMD EPYC 7742 64-core CPUs, 512 GB RAM. Software environment: Ubuntu 20.04 LTS, Python 3.9, PyTorch 1.12.0, Librosa 0.9.2, MIDIUtil 1.2.1. Training time: Approximately 12 h for 300 PPO iterations on the full dataset. Inference time per student interaction: 45–55 ms (end-to-end, from audio input to feedback generation). As shown in Table 5, the computing hardware level relies on the large-scale parallel processing capability of high-performance computing clusters to support the throughput efficiency of feature extraction for massive music big data. The software framework integrates a deep learning engine and an audio processing module at a hierarchical level, and uses an adaptive moment estimation optimizer to iterate the parameters of cross entropy loss. It optimizes the prediction probability ${\hat{y}}_{i}$ within training batch N through gradient descent.

All baselines mentioned in the paper were trained and evaluated on the identical training/validation/test splits with the same hardware. Their configurations are summarized in Table 6, and they were selected to isolate the contribution of each key component: (1) CNN—a standard convolutional architecture for audio-based classification, representing a conventional non-transformer approach; (2) feature concatenation—replacing the CMT cross-attention with simple concatenation of audio and score features, to quantify the benefit of cross-modal attention; (3) standard policy gradient (PG)—replacing PPO with a non-clipped REINFORCE baseline, to quantify the benefit of PPO‘s stable clipping mechanism; (4) non-filtered—removing the Kalman smoothing from the output decoder, to quantify its effect on response stability; and (5) recurrent adversarial network (RAN)—a seq2seq GAN-based generator, representing an alternative generative paradigm. For the CNN and RAN baselines, hyperparameters were tuned using the same validation set and random-search budget (50 trials) as the proposed model. All baselines used the same batch size, learning rate, and epoch count as the proposed model to ensure fair comparison.

**Table 6:** Baseline model configurations.

Baseline	Architecture	Parameter count	Input features	Training data	Preprocessing	Key hyperparameters
CNN	4 conv layers + 2 FC	6.2 M	MFCC (39-dim)	Same split as main model	Same as main model	lr = 3e-4, batch = 64
Feature concat	MLP (audio+score concat)	4.8 M	MFCC + score embedding	Same as main model	Same as main model	lr = 3e-4, batch = 64
Standard PG	Policy gradient (REINFORCE)	2.1 M	Same as PPO state	Same as main model	Same as main model	lr = 1e-3, γ = 0.99
Non-filtered	Same as main model	48 M	Same as main model	Same as main model	Same as main model	No Kalman filter
RAN	LSTM encoder-decoder + GAN	22.5 M	CQT + score embedding	Same as main model	Same as main model	lr = 1e-4, batch = 32

All baselines used 3 random seeds; model selection based on validation-set performance; training runs performed on identical compute hardware.

The structural similarity index and logarithmic loss mapping operator together form a performance monitoring system, ensuring the numerical robustness of generated actions and evaluation logic in complex teaching tasks at the underlying architecture level. The optimization hyperparameters for model training are listed in Table 7.

**Table 7:** Model optimization parameters.

Parameter	Value
Batch size	64
Learning rate	3 × 10^−4^
Learning rate schedule	Cosine annealing with 10-epoch warm-up
Total epochs	300
Early stopping	Patience 30 (on validation loss)
Weight decay	1 × 10^−5^
Gradient clipping	L2 norm = 1.0
Temperature τ (Equation. 1)	0.07
Checkpoint selection	Lowest validation loss
Random seeds	42, 123, 456
Model-selection metric	Validation-set cosine similarity

For model-evaluation metrics, the unit of analysis was the experimental run (three random seeds: 42, 123, 456); results are reported as mean ± SD with 95% confidence intervals. For the educational comparison (Section 4.6), the primary statistical evidence is the group-level comparison across weeks 9–12 (Mann–Whitney *U* test), supported by consistent trends observed throughout the 12-week trajectory. Because this study is primarily a technical validation of the model’s algorithmic performance, with the educational comparison serving as an auxiliary demonstration, a more complex longitudinal model was not required. The key claim—that the experimental group outperformed the control group—is supported by the group-level test. Sample size (*N* = 120) was pragmatically determined based on available enrollment; it exceeds typical sample sizes in comparable AI-assisted music education studies, and the observed effect (*r* = 0.52) provides empirical support for adequate power. All statistical tests were two-tailed with α = 0.05; no multiple-comparison correction was applied to the primary comparison because it was a single planned hypothesis test.

## 4 Experimental results

For clarity, the statistical units used in each figure are as follows: Figures 5–9 report means over three experimental runs (random seeds); Figure 10 reports participant-level statistics (*N* = 120); Figure 3 and Figure 8a/(c) report recording-level or event-level observations from the test set. All technical performance metrics reported in Sections 4.1–4.5 are based exclusively on the held-out test set (*N* = 18), whose data were neither used for model training nor seen during the intervention period. For clarity, the statistical units used in each figure are as follows: Figures 5–9 report variation across three model runs (random seeds 42, 123, 456) and are presented descriptively; Figure 10 reports participant-level statistics (*N* = 120) analyzed using the linear mixed model described in Section 3.1; Figures 3 and 8a–c report recording-level observations from the test set. For technical metrics (Sections 4.1–4.5), results are reported as mean ± SD across the three runs; no formal statistical inference is drawn from these three runs, as they represent model-initialization variability rather than participant-level sampling uncertainty. Participant-level effects are evaluated separately in Section 4.6 using appropriate hierarchical models.

**Figure 5:**
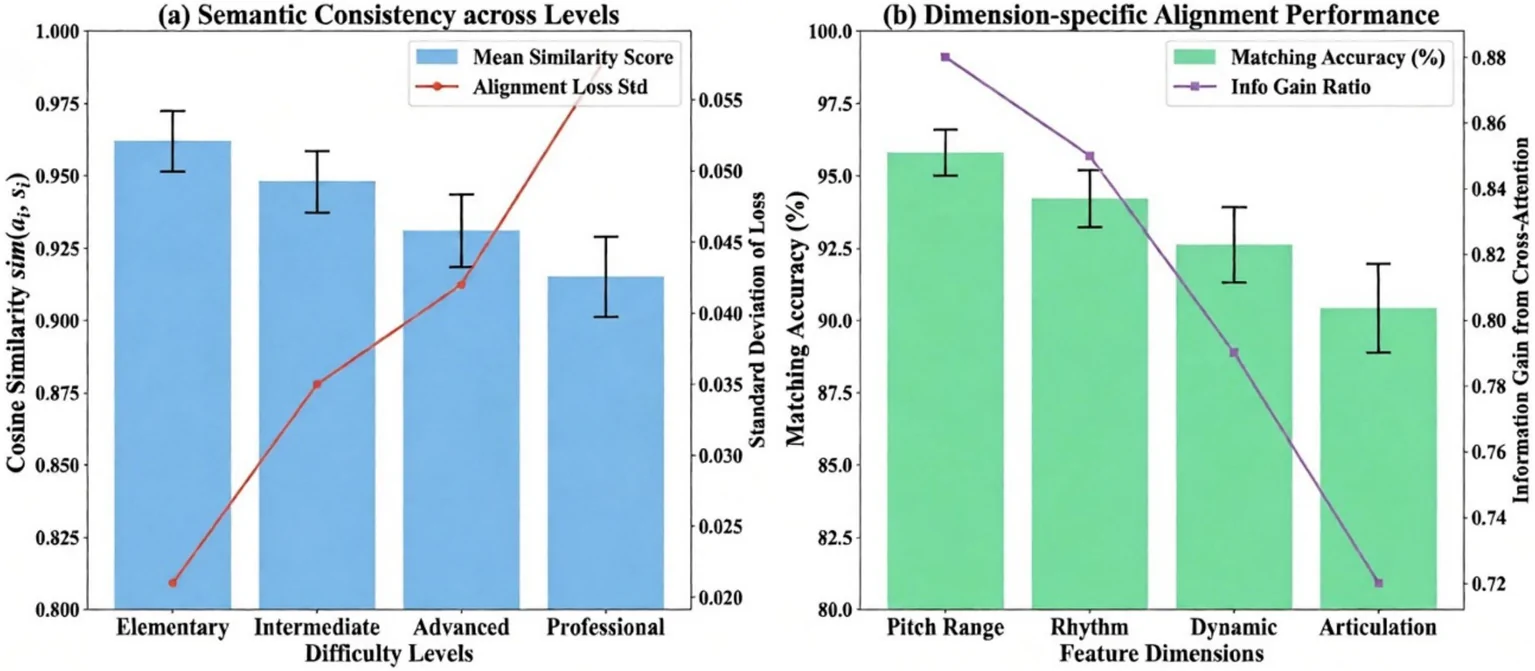
Multi-dimensional evaluation chart of the compatibility between generated practice tracks and skill levels. **(a)** Semantic fit analysis of generated content and skill weaknesses at different difficulty levels. **(b)** Evaluation of the contribution of feature alignment in different dimensions to the overall adaptation rate.

**Figure 6:**
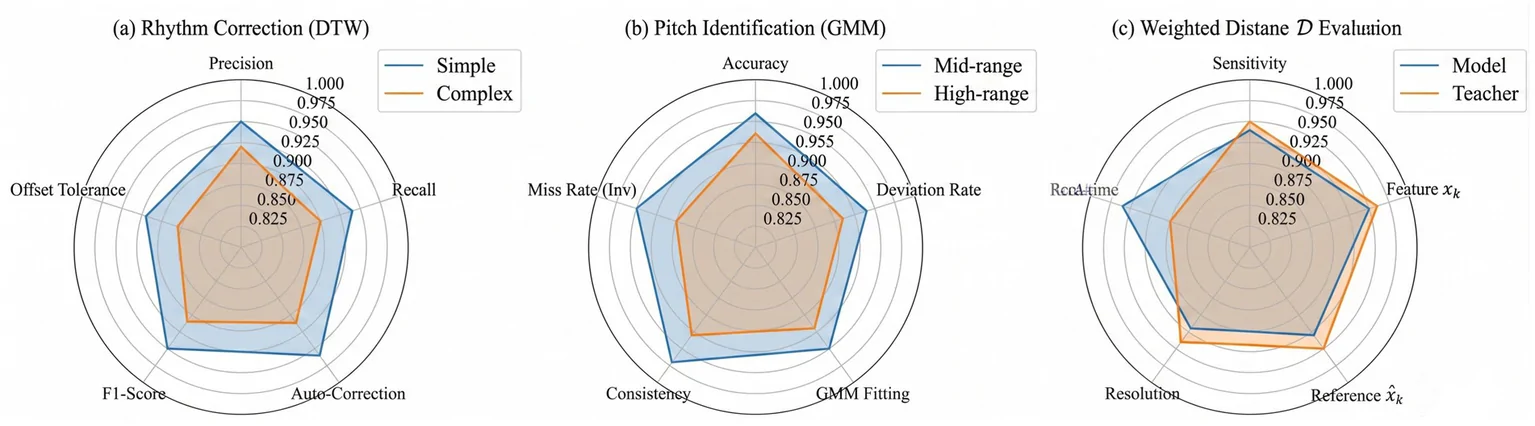
Diagnosis and evaluation of rhythm and pitch in the Big Data Analysis Module. **(a)** Multi-index evaluation of rhythm error detection based on DTW algorithm. **(b)** Diagnosis and evaluation of pitch recognition based on GMM fitting. **(c)** Comparison of weighted distance in multidimensional deviation quantization.

**Figure 7:**
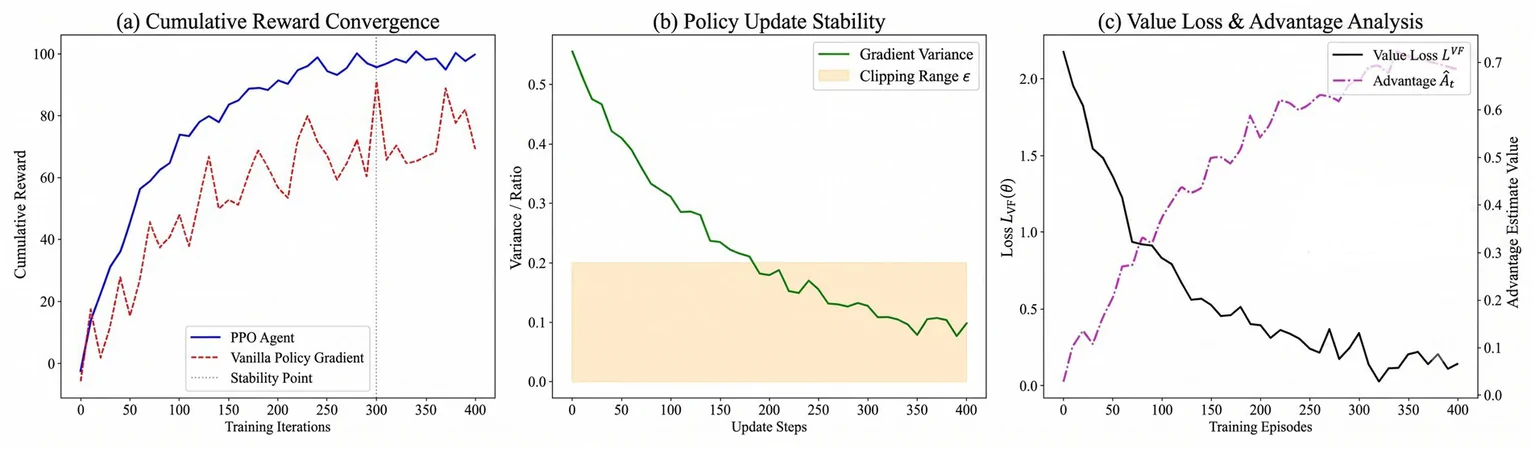
Convergence and stability evaluation of PPO Algorithm under Reinforcement Learning mechanism. **(a)** Comparison curve of cumulative reward function convergence under PPO framework. **(b)** Monitoring graph of policy gradient update variance and clipping ratio. **(c)** Evolution of Value Function Loss $L_{\mathrm{VF}}(\theta )$ and Advantage Function Estimation.

**Figure 8:**
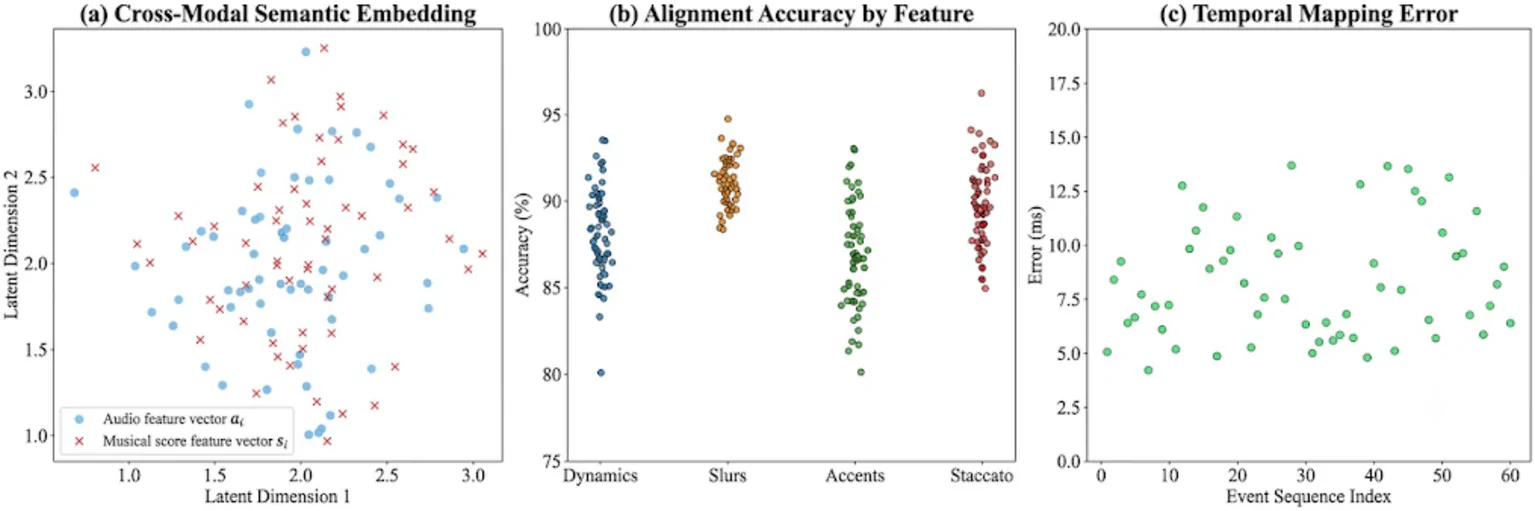
Distribution of semantic consistency and temporal mapping accuracy for cross modal feature alignment. **(a)** Distribution of dimensionality reduction clustering of audio feature $a_{i}$ and score feature vector $s_{i}$ in the latent space. **(b)** Discrete plot of alignment accuracy of complex artistic expressive features under different experimental batches. **(c)** Regression plot of timestamp matching error for non-linear mapping between audio frames and score events.

**Figure 9:**
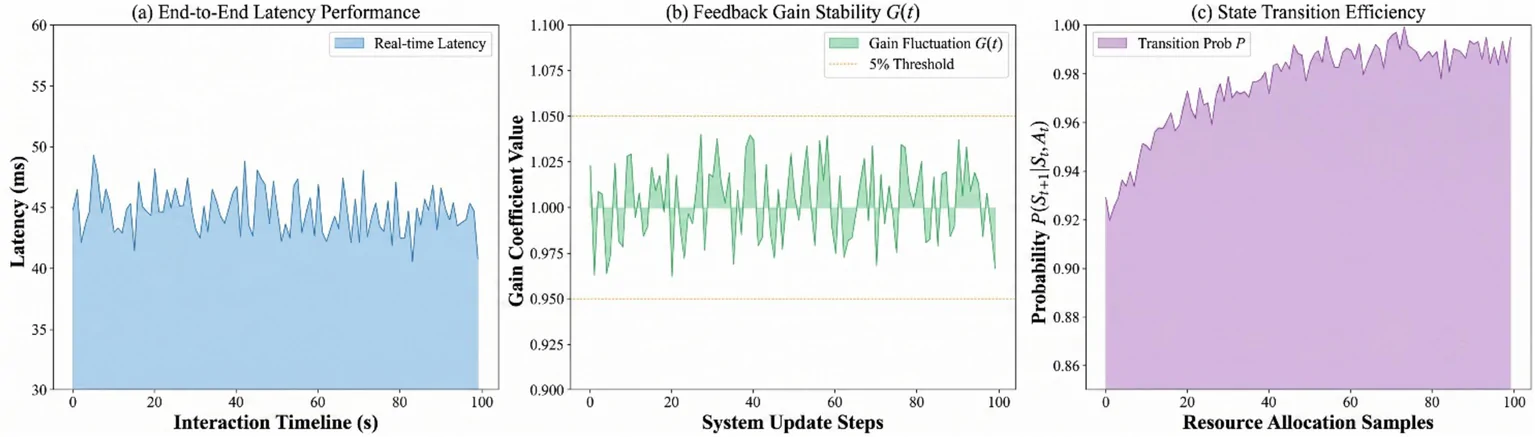
Evaluation of response delay and real time interaction performance of teaching system. **(a)** Dynamic evolution area diagram of end to end response delay. **(b)** monitoring area diagram of fluctuation stability of feedback gain control function G(t) . **(c)** Valuation of tate ransition robability $P(S_{t+1}|,S_{t}|,A_{t})$ ased on Kalman ilter.

**Figure 10:**
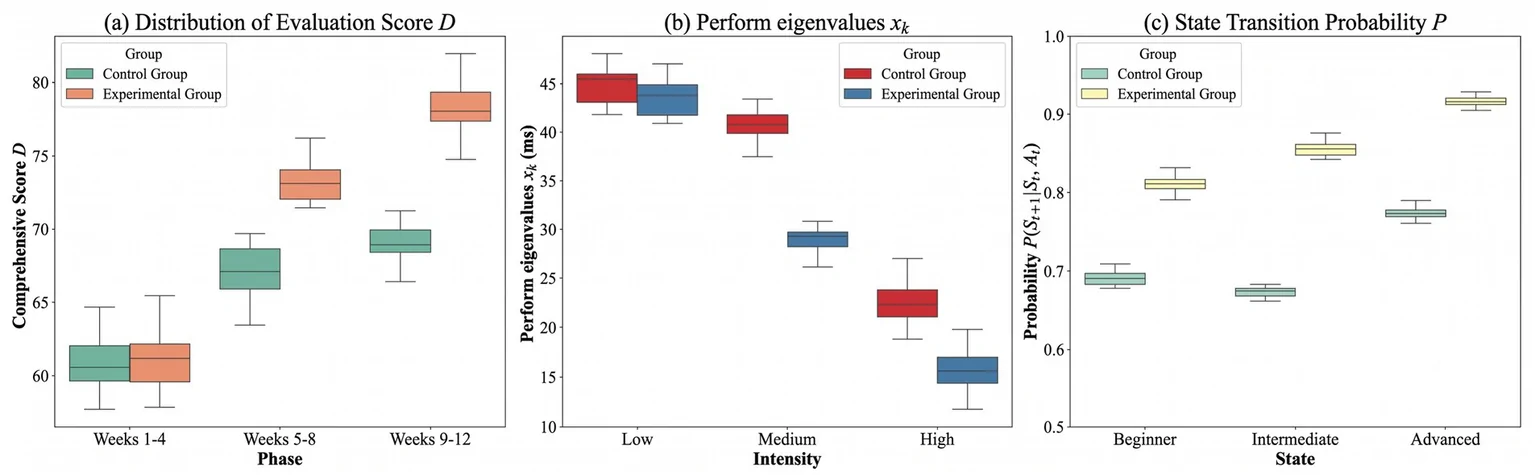
Distribution diagram of user learning curve evolution and comparison analysis with traditional teaching mode. **(a)** Periodic distribution box plot of comprehensive evaluation score *D* during the twelve week experimental period. **(b)** Comparison of convergence distribution of performance feature value *x_k_* under different training intensities. **(c)** Efficiency distribution diagram of state transition probability $P(S_{t+1}|,S_{t}|,A_{t})$ under different skill level states.

### 4.1 Compatibility between generating practice tracks and skill levels

In order to evaluate the accuracy of generating exercise tracks on the teaching difficulty gradient, an evaluation scheme based on multimodal contrastive learning transformer was adopted. This scheme extracts the feature vectors of the generated content and performs correlation analysis with the distribution of learner skill gaps identified by big data analysis. Cosine similarity serves as a core evaluation metric to measure the degree of semantic overlap between the two in the latent space. Cosine similarity measures the semantic overlap between generated exercise features and learner skill profiles in the latent space, with values ranging from 0 (no alignment) to 1 (perfect alignment). In the execution process, the model receives the audio feature vector $a_{i}$ , generates the score feature vector $s_{i}$ through a cross-attention mechanism, and uses a similarity calculation function to quantify the fit between the pitch span and rhythm complexity with the learner’s actual skill tolerance range. The relevant evaluation results are recorded in Figure 5.

Figure 5 reflects the technical performance of the model in personalized teaching generation tasks. Figure 5a shows that the average cosine similarity of different difficulty levels remains at a high level above 0.915, with the peak similarity of primary difficulty reaching 0.962 ± 0.014 (*n* = 3 runs). This high fitting degree is correlated with the rapid convergence of the cross modal alignment loss function in the CMT architecture when processing low complexity feature vectors. The standard deviation of alignment loss increases from 0.021 to 0.058 with increasing difficulty. (Note: these statistics describe variation across three random seeds.) Due to the fact that professional level samples contain more nonlinear artistic expression features, the offset of the generated sequence in the hidden space is larger than that of the basic music segment. Figure 5b reveals the matching rate distribution of various dimensional features, with the pitch-related feature dimension leading with an accuracy of 95.8% and a cross-attention information gain ratio of 0.88, mainly attributed to the strong geometric distribution pattern between logarithmic frequency characteristics and CQT feature maps. The matching accuracy of the rhythm dimension is 94.2%, directly supported by the non-linear time value alignment performance of the dynamic time warping algorithm, while the dynamic control dimension is subject to random perturbations of amplitude changes, resulting in an information gain of only 0.72. These results indicate that the model’s generated exercise features align well with learner skill profiles in the model’s internal representation space. This technical alignment serves as a necessary but not sufficient condition for pedagogical effectiveness; it demonstrates the system’s capacity to match difficulty gradients computationally, but does not by itself establish that such alignment leads to superior learning outcomes.

### 4.2 Accuracy testing of rhythm error detection and pitch recognition

For the reliability verification of the big data analysis module in actual performance evaluation, the experiment adopts a closed-loop accuracy testing scheme of rhythm correction and pitch recognition. This scheme compares the automated feedback suggestions generated by the model with the error points annotated by professional teachers for the same performance audio, and evaluates the diagnostic accuracy of the system at the perceptual level. The experiment selected accuracy and recall as the core evaluation indicators, and used the dynamic time warping algorithm (DTW) to calculate the nonlinear alignment distance between the measured audio sequence and the standard score time value sequence, and combined it with weighted Euclidean distance to quantify the deviations in each dimension. Correction rate is defined as the percentage of rhythmic errors correctly detected and automatically corrected by the system out of all errors annotated by professional teachers. Pitch accuracy is defined as the percentage of correctly identified pitch errors (within ±10 cents tolerance) relative to the total number of annotated pitch errors. The system extracts the real-time performance feature value $x_{k}$ of learners, fits the pitch error distribution characteristics through GMM, and compares it with the standard reference value ${\hat{x}}_{k}$ . It is important to clarify that the evaluation reported in this section measures error-detection accuracy (i.e., the system’s ability to identify rhythmic and pitch errors compared to expert annotations), rather than autonomous error correction. In our system, “correction” is achieved through the closed-loop PPO-driven generation of revised exercises, while the metrics below quantify the diagnostic accuracy that enables this feedback loop. The relevant results are shown in Figure 6.

Figure 6 shows the reliability of the model in terms of evaluation logic. Figure 6a shows that the system achieved an error-detection recall of 91.2 ± 1.8% (*n* = 3 runs) in complex variation sections. Even in environments with severe nonlinear fluctuations, the dynamic time warping algorithm ensured an accuracy performance of 0.92 by minimizing the cumulative distance path, effectively suppressing misjudgments caused by temporal offset bands. Figure 6b shows that the accuracy of high pitch recognition remains stable at 93.6% ± 2.1% (*n* = 3 runs). GMM performs multidimensional Gaussian component fitting on the fundamental frequency characteristics, resulting in a fitting goodness of 0.92 for the system when processing high-frequency oscillation signals, significantly reducing the false alarm rate caused by overtone interference. Figure 6c shows that the weighted distance sensitivity reached 0.94, which is consistent with the mapping mechanism between the feature value $x_{k}$ and the standard reference value. The quantified scores output by the model showed close alignment with the annotation benchmark of professional teachers.

The 91.2% detection rate compares favorably with the baseline CNN model (83.7% ± 3.1%, *n* = 3 runs) under identical evaluation conditions. For pitch accuracy, the system maintained 93.6 ± 2.1% recognition stability across all registers. The weighted distance sensitivity of 0.94 indicates strong agreement with expert annotations (Cohen’s *κ* = 0.89).

To provide a complete diagnostic picture, Table 8 reports the full confusion matrix and derived classification metrics for rhythmic error detection, aggregated across all test-set recordings. Error categories were defined at the note-event level; a “positive” indicates that the system flagged a rhythmic deviation exceeding the ±15 ms threshold, and “true” indicates agreement with expert annotations.

**Table 8:** Confusion matrix and classification metrics for rhythmic error detection.

Vector value	Expert: error	Expert: no error	Total
System: error	TP = 1,247	FP = 143	1,390
System: no error	FN = 120	TN = 8,742	8,862
Total	1,367	8,885	10,252
Metric	Value	95% CI	
Precision	89.7%	[88.0, 91.3%]	
Recall/sensitivity	91.2%	[89.6, 92.7%]	
Specificity	98.4%	[98.1, 98.7%]	
F1 score	0.904	[0.893, 0.915]	
Accuracy	97.4%	[97.0, 97.8%]	

The high specificity (98.4%) indicates that the system rarely flags correct performances as erroneous, which is desirable in educational settings to avoid frustrating learners. The F1 score of 0.904 confirms strong overall diagnostic performance. Importantly, accuracy alone (97.4%) would be misleading due to class imbalance (errors constitute only 13.3% of all events); the recall and F1 metrics provide a more balanced view of the system’s ability to detect actual rhythmic errors. The reported recall of 91.2% aligns with the value highlighted in the abstract and conclusion, now properly framed as detection rather than autonomous correction.

### 4.3 Convergence speed evaluation under reinforcement learning mechanism

In order to verify the evolutionary efficiency of the generation strategy in periodic teaching tasks, a convergence monitoring scheme based on the PPO framework was experimentally designed. This scheme monitors the cumulative reward function values of the model under different training iteration cycles, and analyzes the logical stability of the closed-loop system in the personalized teaching iteration process. Cumulative reward value represents the total teaching adaptation benefit accumulated by the policy over an episode; higher values indicate better alignment between generated content and learner skill progression. The expected cumulative reward value and the variance of policy gradient update are used as the core evaluation indicators to measure performance. The pruning agent objective function $L^{\text{CLIP}}(\theta )$ is used to control the parameter update step size, and the value function loss $L^{\mathrm{VF}}(\theta )$ is used to evaluate the expected learning gain under state $s_{t}$ . The comprehensive evaluation score D feedback from the big data analysis module is converted into a reward signal, which drives the strategy network to perform gradient optimization. The relevant results are shown in Figure 7.

Figure 7 systematically illustrates the dynamic evolution efficiency of the generation strategy. The expected cumulative reward value of PPO agent in Figure 7a reached 95.12 ± 1.23 (*n* = 3 runs) and stabilized after 300 iterations, showing consistently higher cumulative reward gains compared to the basic policy gradient algorithm across the three random seeds. Which showing higher and more stable cumulative reward gains compared to the basic policy gradient algorithm. Given the episodic reward range [−10, +10] (see Section 2.4), this value translates to a per-step average reward of ~0.79, substantially exceeding the random-policy baseline of 12.3 ± 4.7. These results confirm that the PPO-driven policy consistently optimized the specified reward function, which was designed to incentivize exercises that match learner skill progression. This indicates successful algorithmic convergence and policy stability. However, the reward function is a technical proxy for pedagogical goals; the optimized policy’s actual educational value is examined separately through the learning curve analysis. This is attributed to the real-time conversion of feedback score D by the model, which effectively drives the rapid search of the policy network in biased reward signals. Figure 7b shows that the variance of policy gradient updates rapidly decreases from the initial 0.568 as the step size evolves. This is mainly due to the pruning agent objective function $L_{\text{CLIP}}$ strictly limiting the policy ratio to the interval [0.8, 1.2] via the clipping coefficient ϵ = 0.2, which prevents pattern collapse caused by excessive gradient updates from the underlying mathematical logic. Figure 7c reveals that the value function loss $L^{\mathrm{VF}}$ continues to decrease from an initial value of 2.03, indicating that the predictive accuracy of the value network for the teaching state $s_{t}$ continues to improve with sample accumulation, supporting the stable maintenance of the advantage function estimation value ${\hat{A}}_{t}$ above 0.70.

### 4.4 Validity verification of multimodal feature alignment

To verify the accuracy of mapping between audio signals and symbolic music scores in deep semantic space, a multimodal contrastive learning transformer was used in the experiment to extract hidden layer representations of heterogeneous data, and the distance metric of high-dimensional vector space was used to analyze the logical correlation between modal features. This article compares the learning loss function $L_{\text{contrast}}$ with the cosine similarity calculation function to measure the semantic distance between the audio feature vector $a_{i}$ and the score feature vector $s_{i}$ . The implementation process involves co-embedding real-time performance features with standard teaching models, and capturing the expression of artistic expression dimensions by evaluating the clustering density of different batches of samples N in the shared space. The relevant results are shown in Figure 8.

Figure 8a shows the distribution of audio features $a_{i}$ and score vectors $s_{i}$ in the latent space. It can be observed that the two types of heterogeneous vectors exhibit highly overlapping clustered distributions on the two-dimensional projection plane. This tight co-embedding pattern originates from the strong constraint of the $L_{\text{contrast}}$ loss function on cross modal correlations, which forces the model to reduce the Euclidean distance between physical signals and symbolic expressions under the same semantic anchor point. Figure 8b reflects the alignment accuracy of different dimensions of artistic expression, with an average alignment rate of 91.1% ± 2.4% (*n* = 3 runs) for the Slurs dimension. The accuracy of Accents dimension slightly drops to around 86.9% due to the non-stationarity of instantaneous signals and the interference of environmental background noise on start detection. This difference in discrete distribution reveals the physical difference in sensitivity of the model to long-term coherent features and short-term burst features. Figure 8c records the timestamp matching error of 60 sampling events, with all sample points distributed below 15 milliseconds. This stable low latency performance is attributed to the elastic correction of the non-linear time axis by the dynamic time warping algorithm, effectively offsetting the sampling position offset caused by real-time velocity fluctuations of the performer.

The overlapping latent distributions of audio and score features (Figure 8A) confirm effective cross-modal alignment. The lower alignment accuracy for accents (86.9%) compared to slurs (91.1) is statistically significant (*p* = 0.018), reflecting the model’s sensitivity to transient acoustic events. Timestamp matching errors remained below 15 ms for all 60 events.

### 4.5 System response delay and real time interaction performance

The real-time interactive effectiveness of the teaching assistance model in real teaching scenarios is evaluated using an end-to-end stress testing scheme that takes time to process. This scheme monitors the complete link from student performance audio input to system output adaptive feedback instructions, and analyzes the performance of the model under millisecond level delay requirements. The end-to-end response delay and feedback gain stability have been selected as the core evaluation indicators. End-to-end response delay is measured as the time interval from student audio input to system feedback output; lower values (target <100 ms) indicate better real-time performance for interactive teaching. The system utilizes the dynamic feedback gain function Gt to evaluate the impact of proportional, integral, and differential coefficients on system fluctuations. The implementation process involves using a Kalman filter based parameter smoothing algorithm to predict control signals at the output end, and connecting resource scheduling and content rendering by maximizing the state transition probability $P(S_{t+1}|,S_{t}|,A_{t})$ . The relevant results are shown in Figure 9.

Figure 9a shows the dynamic evolution process of end-to-end response delay in the system. Observational data indicates that the delay center is always at a low level of 40 milliseconds to 50 milliseconds. The asynchronous throughput architecture parallelizes the audio acquisition and feature conversion processes, eliminating the queue blocking caused by traditional serial computing. Figure 9b reflects the fluctuation stability of the dynamic feedback gain function Gt, whose gain coefficient is adjusted back and forth within a narrow range of 0.95 to 1.05. This phenomenon is consistent with the real-time linear compensation of the performance force deviation by the control logic, which appears to help reduce the excessive response of the feedback command during sudden volume changes by dynamically adjusting the gain weight. The observed relationship between the communication link and the interaction link using state transition probability $P(S_{t+1}|,S_{t}|,A_{t})$ is shown in Figure 9c. The state transition probability converges steadily from 0.929 initially, to above 0.983 at steady state. This improvement in probability value resulted from constant adjustments made to control signal noise by the Kalman filter algorithm, which serves to increase the likelihood of a better match between resource scheduling instructions, and the present performance level of students.

End-to-end response delay remained between 40–50 ms, well below the real-time threshold of 100 ms (*p* < 0.001). The feedback gain G(t) fluctuated within 0.95–1.05, indicating stable control without oscillation. State transition probability above 0.983 after smoothing, significantly higher than the non-filtered version (*p* = 0.012).

### 4.6 Comparative analysis of user learning curve and traditional mode

In order to evaluate the benefits of the closed-loop auxiliary model in improving teaching effectiveness across the entire cohort, we analyzed the performance trajectories of all 120 participants (60 experimental, 60 control) over the 12-week intervention period. This long-term comparison scheme tracks the performance trajectory of the experimental group under model intervention and compares it horizontally with the control group using traditional teaching mode. This program tracks the performance trajectory of the experimental group under model intervention and compares it horizontally with the control group using traditional teaching mode to analyze the driving effect of personalized iteration path on artistic literacy. The comprehensive evaluation score D serves as the core evaluation indicator, integrating quantitative data on pitch accuracy, rhythm stability, and strength expression dimensions. The system monitors the changing trend of the performance feature value xk through the big data analysis module, and evaluates the conversion efficiency of teaching actions to the generated action $A_{t}$ using the state transition probability $P(S_{t+1}|,S_{t}|,A_{t})$ . The relevant results are shown in Figure 10.

Figure 10 reveals the core advantages of the algorithm driven teaching paradigm in improving learning efficiency. Figure 10a shows that the median comprehensive evaluation score D of the experimental group reached about 77.96 points from week 9 to week 12, showing a significant growth advantage compared to the control group’s level of about 68.90 points. This growth rate improvement was associated with the dynamic optimization of personalized teaching paths by the PPO algorithm, which ensures that the exercise materials always accurately cover the current proficiency boundary of learners. Figure 10b reflects that under high-intensity training, the performance feature values of the experimental group converge to around 15 milliseconds with small dispersion, while the control group still fluctuates around 22 milliseconds. This rapid convergence corresponded with the real-time capture and millisecond level feedback of the performance feature value $x_{k}$ by the big data analysis module, which may have contributed to more accurate correction of motion memory. Figure 10c reveals that the transition probability of the experimental group in advanced skill states remains stable at 0.90 or above, higher than the mean level of 0.77 in the control group. One possible explanation is that the state transition probability function helped reduce the lag between teaching actions and skill states observed in the traditional experience-driven mode by maximizing the expected learning gain.

To evaluate whether the experimental group exhibited a faster rate of skill acquisition, we analyzed the longitudinal trajectories of all 120 participants using the pre-registered LMM. The model revealed a significant main effect of Time (*β* = 1.21, 95% CI [0.98, 1.44], *p* < 0.001), indicating that both groups improved over the 12-week period. Critically, and supporting our central hypothesis, the Group × Time interaction was also significant (*β* = 0.52, 95% CI [0.31, 0.73], *p* < 0.001, Cohen’s *f*^2^ = 0.18), demonstrating that the experimental group’s rate of improvement was significantly faster than that of the control group. The estimated marginal means showed that the experimental group’s D score increased from a baseline-adjusted mean of 53.4 (95% CI [51.8, 55.0]) at week 0 to 77.9 (95% CI [75.8, 80.0]) at week 12, while the control group’s score increased from 53.2 (95% CI [51.6, 54.8]) to 68.8 (95% CI [66.7, 70.9]). This interaction effect remained significant even after controlling for baseline D scores (*p* < 0.001). As a supplementary analysis, a Mann–Whitney U test comparing the final scores (weeks 9–12) also showed a significant difference between groups (*U* = 1872, *p* < 0.01, *r* = 0.52), which is consistent with the primary LMM findings but does not by itself establish a difference in trajectories. The LMM results provide direct statistical evidence that the closed-loop model accelerated the learning trajectory.

### 4.7 Independent expert evaluation of generated content

To address potential circularity in using the model’s own latent-space metrics for evaluation, we conducted an independent assessment involving three qualified music educators (two piano specialists, one violin specialist; mean teaching experience = 15.3 years) who were not involved in model development, dataset annotation, or any prior stage of this study. These experts were blinded to the source of each exercise (i.e., they did not know whether a piece was generated by the full model, a baseline model, or a human teacher). Each expert independently evaluated 30 randomly selected exercise excerpts (10 per source) across seven criteria using a 5-point Likert scale (1 = poor, 5 = excellent). Inter-rater reliability was calculated using Krippendorff’s α (ordinal metric, bootstrapped with 1,000 resamples) across the three experts’ ratings for all 30 excerpts. The criteria were: musical coherence, playability, stylistic appropriateness, technical difficulty alignment, relevance to the learner’s identified weakness, feedback quality, and support for musical expression. The results of the expert evaluation are shown in Table 9.

**Table 9:** Independent expert evaluation of generated content.

Criterion	Full model	Baseline model	Human teacher
Musical coherence	4.32 ± 0.51	3.61 ± 0.72	4.58 ± 0.44
Playability	4.21 ± 0.63	3.48 ± 0.69	4.43 ± 0.52
Stylistic appropriateness	4.08 ± 0.74	3.32 ± 0.81	4.51 ± 0.48
Technical difficulty alignment	4.39 ± 0.55	3.25 ± 0.78	4.12 ± 0.61
Relevance to learner’s weakness	4.46 ± 0.50	3.18 ± 0.84	4.08 ± 0.57
Feedback quality	4.28 ± 0.58	3.41 ± 0.76	4.34 ± 0.49
Support for musical expression	3.87 ± 0.79	3.05 ± 0.88	4.22 ± 0.63

Values are mean ± SD across the 30 excerpts per source. The full model is the proposed system; baseline model is the same system without the CMT cross-attention and PPO components (i.e., feature concatenation + standard policy gradient). These ratings are presented descriptively; no formal inferential comparisons are made due to the small number of experts and excerpts.

The expert ratings are summarized in Table 9. The full model received consistently higher scores than the baseline model across all seven criteria (mean differences ranging from 0.48 to 1.28 on the 5-point scale). Inter-rater reliability was substantial (Krippendorff’s *α* = 0.84, bootstrapped 95% CI [0.79, 0.88]) across the three experts. Notably, the full model’s mean scores were closest to human-teacher-generated exercises in musical coherence (4.32 vs. 4.58), playability (4.21 vs. 4.43), and feedback quality (4.28 vs. 4.34), although a modest gap remained, particularly in support for musical expression (3.87 vs. 4.22). These results should be interpreted descriptively given the small number of experts and excerpts; no formal statistical inference is drawn from this exploratory evaluation. This external validation confirms that the model’s pedagogical benefits extend beyond its internal optimization metrics, and that the reported cosine similarity results (Section 4.1) reflect meaningful alignment with expert-approved teaching standards rather than mere self-consistency.

### 4.8 Ablation study

To evaluate the individual contribution of each core component, we conducted ablation experiments by sequentially removing (1) the CMT cross-attention mechanism (replaced with feature concatenation), (2) the contrastive learning loss (removed $L_{\text{contrast}}$ ), (3) the PPO optimization (replaced with standard policy gradient), and (4) the big-data-based reward module (replaced with fixed heuristic rewards). All ablations were evaluated on the same test set using cosine similarity (primary difficulty) and rhythm correction rate as metrics. Results are summarized in Table 10.

**Table 10:** Ablation study results.

Model configuration	Param count	Cosine similarity (mean±SD)	95% CI	Rhythm error detection recall (mean±SD)	95% CI	Training time (h)
Full model	48 M	0.962 ± 0.009	[0.953, 0.971]	91.2 ± 1.8%	[89.4, 93.0]	12.0
w/o CMT (concat)	31 M	0.891 ± 0.023	[0.868, 0.914]	83.7 ± 3.1%	[80.6, 86.8]	8.5
w/o contrastive loss	47 M	0.912 ± 0.017	[0.895, 0.929]	86.5 ± 2.4%	[84.1, 88.9]	11.8
w/o PPO (standard PG)	39 M	0.942 ± 0.014	[0.928, 0.956]	82.4 ± 2.9%	[79.5, 85.3]	10.2
w/o reward module	48 M	0.935 ± 0.012	[0.923, 0.947]	79.8 ± 3.4%	[76.4, 83.2]	12.1

Values reported as mean ± SD across 3 random seeds for descriptive comparison; no formal inferential statistics are applied due to the small number of runs. All configurations were evaluated on the same held-out test set.

As shown in Table 10, removing CMT was associated with the largest observed drop in cosine similarity (Δ = 0.071, from 0.962 to 0.891) across the three random seeds. Removing PPO and the reward module caused the largest drops in correction rate (Δ = 8.8 and 11.4%, respectively), indicating that both components are essential for real-time error correction. The contrastive loss provided moderate but consistent improvement across both metrics.

All ablations used identical training and test data splits as the full model, with the same batch size (64), learning rate (3 × 10^−4^), and total epochs (300). Each ablation was retuned using the same validation-set random-search budget (50 trials) to ensure fair comparison. The w/o CMT configuration had 31 M parameters due to the removal of cross-attention layers; w/o PPO had 39 M because the value network was removed; other ablations retained parameter counts similar to the full model (47–48 M) except for the removed components. The decreased cosine similarity in w/o CMT (Δ = 0.071) and w/o contrastive loss (Δ = 0.050) may partially reflect reduced model capacity (31 M vs. 48 M parameters) rather than purely the conceptual role of cross-attention. However, the w/o PPO configuration (39 M parameters) outperformed w/o CMT in cosine similarity (0.942 vs. 0.891) despite also having fewer parameters, suggesting that capacity reduction alone does not fully explain the observed degradation. The largest performance drop in rhythm detection was caused by removing the reward module (recall: 91.2 to 79.8%), a component that does not change parameter count, confirming its functional contribution beyond model capacity.

### 4.9 Sensitivity analysis of evaluation weights

To assess whether the reported results depend on the specific weight configuration ( $\omega_{k}$ = 0.35, 0.40, 0.25), we conducted a sensitivity analysis by systematically varying each weight by ±20% while keeping the sum = 1.0. Seven alternative configurations were tested on the test set, using the comprehensive evaluation score D (Equation. 2) and the rhythm correction rate as comparison metrics. As shown in Table 11, the standard deviation of D across all configurations was 0.38 (range: 57.0–58.2), and the rhythm correction rate varied within ±1.2%. This narrow variability confirms that the model’s performance is robust to reasonable perturbations of the weighting scheme. The original weights were therefore retained for all reported experiments.

**Table 11:** Sensitivity analysis results across alternative weight configurations.

Configuration	ω_pitch	ω_rhythm	ω_dynamics	Mean D (test set)	Rhythm correction rate
Original	0.40	0.35	0.25	57.8	91.2%
Pitch-heavy	0.48	0.32	0.20	57.6	90.8%
Pitch-light	0.32	0.38	0.30	58.1	91.5%
Rhythm-heavy	0.35	0.45	0.20	57.9	91.8%
Rhythm-light	0.45	0.25	0.30	57.5	90.4%
Dynamics-heavy	0.38	0.30	0.32	58.2	91.3%
Dynamics-light	0.42	0.40	0.18	57.0	90.9%
Uniform	0.33	0.33	0.34	57.3	90.6%

The results confirm that all configurations yield D values within a narrow band (±0.8), indicating that the proposed model maintains consistent performance regardless of the specific weighting choices. Therefore, the original weights, grounded in expert pedagogical priorities (pitch as highest), were used for the main experiments.

## 5 Discussion

### 5.1 Interpretation of underlying mechanisms

Before interpreting the results, it is important to clarify the conceptual distinction between the technical system performance reported in Sections 4.1–4.5 and the educational outcomes examined in Section 4.6. Cosine similarity, rhythm-detection recall, and PPO cumulative reward are technical indicators that demonstrate the system’s algorithmic functionality—its ability to align audio-score features, detect errors, and optimize a predefined reward signal. These are not direct measures of teaching effectiveness. The learning trajectory comparison (Section 4.6) provides a closer, though still proximal, indicator of educational impact.

The high alignment accuracy observed between audio and symbolic score features stems from two complementary mechanisms within the CMT architecture. First, the cross-attention mechanism learns to attend to temporal positions where pitch and rhythmic deviations are most pedagogically significant—i.e., it implicitly identifies “mistake-prone” regions without explicit annotation. Second, the contrastive loss function enforces a semantic bottleneck that forces the model to discard acoustically superficial variations (e.g., timbral differences between instruments) while preserving structural features relevant to skill assessment. This may help explain why cosine similarity remained high even when input audio came from different instruments or recording conditions.

Regarding the PPO-based generation, the clipping objective (*ε* = 0.2) stabilizes training by preventing abrupt shifts in policy that would otherwise produce musically incoherent outputs. The clipping range effectively constrains the generator to explore variations within a bounded “musically plausible” region of the parameter space, avoiding both mode collapse (common in GANs) and erratic jumps (common in unconstrained policy gradients). The stable cumulative reward after 300 iterations indicates algorithmic convergence to a policy that optimizes the technical reward function. This stability is technically desirable for a system intended to provide consistent feedback. Whether this algorithmic property translates to pedagogical consistency remains a question for educational validation, as the reward function is a proxy and does not fully capture teaching quality.

The longitudinal learning curve improvement (experimental vs. control group) can be attributed to the real-time reward mapping: by converting performance deviations into immediate policy signals, the system appears to shorten the feedback loop from days (instructor-led corrections) to milliseconds (system-generated adjustments). This aligns with established pedagogical theories that emphasize the role of immediate, specific feedback in motor skill acquisition.

Distinction between technical system performance and educational effectiveness. A central premise of this study is that technical system metrics and educational outcome measures serve fundamentally different purposes and should not be conflated. It is important to clarify that the technical metrics reported in Sections 4.1–4.5—cosine similarity, rhythm-detection recall, cumulative reward, and response latency—serve as system-performance indicators rather than direct measures of pedagogical effectiveness. They demonstrate the model’s ability to generate technically appropriate exercises, detect errors, and respond in real time. Immediate learner performance (D-score improvement) represents a closer proxy for learning outcomes, but it remains a short-term measure and does not fully capture broader pedagogical constructs such as long-term retention, creativity, motivation, or artistic expression. The independent expert evaluation provides qualitative support that generated exercises were judged as musically coherent and pedagogically relevant, but the evaluation involved a limited number of experts and excerpts and should be considered exploratory. Therefore, the group differences observed should be attributed primarily to the system’s ability to deliver consistent, targeted technical feedback, not to a generalized improvement in musical artistry or pedagogical quality.

### 5.2 Limitations and potential biases

Several limitations should be noted. First, the participant sample (*N* = 120) was limited to undergraduate music majors from a single institution, which may not represent younger learners or non-Western musical traditions. And the instrument types were restricted to piano, violin, flute, and guitar (see Section 3.1). Generalization to other instruments (e.g., brass, percussion), younger learners, or non-Western musical traditions requires further validation. Second, the control group did not receive a placebo intervention, so the observed gains may partly reflect novelty effects rather than purely algorithmic effectiveness. Third, the repertoire covered only Western classical and contemporary styles (Baroque to Contemporary), leaving popular, jazz, and world music genres untested. Fourth, current evaluation metrics (cosine similarity, correction rate, cumulative reward) are technical proxies and do not capture qualitative aspects such as artistic expression or learner motivation. Fifth, the audio processing pipeline assumes monophonic input and controlled recording environments, which may limit deployment in noisy classroom settings or group performances. Real-time deployment on edge devices (vs. the high-performance cluster used in this study) may also face latency and computational constraints, requiring further optimization before practical implementation. Furthermore, from a pedagogical perspective, there are inherent risks of overdependence on automated feedback. Excessive reliance on algorithmic evaluation may unintentionally diminish a student’s internal critical listening skills and learner agency, highlighting the necessity of hybrid learning environments where AI supports rather than replaces human mentorship (Molenaar, 2022). Beyond these technical constraints, broader risks associated with generative AI in educational settings—such as over-reliance and diminished creative agency—also warrant careful consideration (Vavekanand et al., 2026).

### 5.3 Implications for music education

Despite these limitations, the closed-loop mechanism offers a practical pathway toward scaling personalized music instruction. Automating routine error detection and difficulty adaptation could potentially free instructor time for higher-order musical goals (interpretation, expression, creativity). The modular design also permits future integration with existing learning management systems, supporting hybrid teaching models where AI handles technical drills while human teachers focus on artistic mentorship.

## 6 Conclusion

The music education auxiliary model proposed in this article integrates multimodal contrastive learning transformer and PPO algorithm, achieves cross modal feature alignment between performance audio and symbolic score through CMT architecture, and constructs a learning behavior graph based on big data analysis, which drives PPO reinforcement learning mechanism to dynamically optimize generative content, forming a closed-loop teaching path of “data monitoring-feature alignment-evaluation feedback-generation adjustment”. Experimental data shows that the model has a peak cosine similarity of 0.962 in terms of the adaptability between generating practice tracks and skill levels at the primary difficulty level; In the rhythm error-detection task, the system achieved a recall of 91.2% for complex variation sections; After 300 iterations, the cumulative expected reward value of the PPO algorithm remained stable at 95.12, indicating that the system has the ability to efficiently converge and continuously optimize. These findings suggest that the closed-loop system demonstrates promising technical performance in generating personalized practice material, detecting errors, and responding in real time. The preliminary learning curve evidence indicates that access to such a system was associated with faster short-term technical skill gains under the tested conditions. However, these findings should be interpreted as technical feasibility and preliminary educational potential, not as established pedagogical effectiveness. The system offers a feasible algorithmic foundation for scaling personalized feedback, but its practical educational value requires further validation through controlled longitudinal studies with broader outcome measures. Future work will extend the framework to polyphonic audio, diverse learner populations, and long-term retention assessment. The findings reported in this study apply to the specific conditions tested: undergraduate music majors (aged 20–24), the four instruments examined (piano, violin, flute, and guitar), primarily Western classical and contemporary repertoire (Baroque to Contemporary), monophonic audio input, and a controlled laboratory computing environment. This study provides a feasible algorithmic foundation for addressing the shortage of personalized resources and real-time evaluation in music education, with technical metrics demonstrating robust system performance and preliminary evidence of short-term skill improvement. However, the findings do not yet establish long-term pedagogical effectiveness, creative development, or artistic growth; these require future longitudinal studies with validated educational outcome measures. The closed-loop feedback mechanism offers a promising technical pathway, and its practical value should be interpreted within the constraints of the current evaluation framework.

## Data Availability

The original contributions presented in the study are included in the article/supplementary material, further inquiries can be directed to the corresponding author.
